# Metabolism‐Based Biomarkers for Rapid Phenotypic Antibiotic Susceptibility Testing

**DOI:** 10.1002/advs.75553

**Published:** 2026-05-07

**Authors:** Sha Yu, Bayinqiaoge, Xi Lu, Xin Wang, Rongfeng Wang, Yi Li, Shi‐Yang Tang, Chengchen Zhang

**Affiliations:** ^1^ Digital Health and Biomedical Engineering School of Electronics and Computer Science University of Southampton Southampton UK; ^2^ School of Mechanical and Manufacturing Engineering University of New South Wales Sydney New South Wales Australia; ^3^ Key Laboratory of Clinical Laboratory Diagnostics (Ministry of Education) College of Laboratory Medicine Chongqing Medical University Chongqing China

**Keywords:** antibiotic susceptibility testing, bacteria, biomarker, metabolism

## Abstract

The accelerating global crisis of antimicrobial resistance (AMR) demands rapid and accurate methods for antibiotic susceptibility testing (AST). Conventional phenotypic assays remain the gold standard but are hindered by long culture times, while genotypic tests cannot reliably predict phenotypic resistance. In recent years, metabolism‐based AST has emerged as a promising alternative, enabling the rapid detection of bacterial responses to antibiotics through shifts in metabolic activity. These approaches bridge molecular speed with phenotypic precision, allowing susceptibility determination within hours, or even minutes, without requiring cell proliferation. In this review, we summarize the latest advances in metabolism‐based biomarkers for rapid AST. First, we discuss how antibiotics influence bacterial metabolism, linking resistance mechanisms to metabolic activities. We then summarize emerging metabolic biomarkers, categorized by their physiological underpinnings: nutrient uptake, respiratory activity, metabolic reprogramming, and enzymatic function. Finally, we list key challenges and future directions toward deployable metabolism‐based AST platforms.

## Graphical abstract

Metabolic differences emerging after antibiotic exposure provide rapid phenotypic signals of susceptibility and resistance. This review summarizes metabolic biomarkers for rapid phenotypic AST, associated with nutrient uptake, respiratory activity, metabolic reprogramming, and enzymatic function.

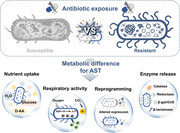

## 1 Introduction

Since their discovery, antibiotics have played a fundamental role in treating bacterial infections, saving millions of lives worldwide. However, the misuse and overuse of these drugs have led to the rapid emergence of antimicrobial resistance (AMR), which is now a growing global concern [1]. The epidemiological projections indicated that AMR could cause up to 10 million deaths per year by 2050 and result in a 2%–3.5% decline in global gross domestic product [2]. One of the most critical strategies to combat AMR is the timely and accurate determination of bacterial susceptibility to antibiotics, known as antibiotic susceptibility testing (AST) [3]. It is essential for guiding appropriate antibiotic use, improving clinical outcomes, and limiting the spread of resistance [4].

Traditional AST methods can be broadly categorized into genotypic and phenotypic approaches. Genotypic methods, using polymerase chain reaction (PCR), DNA microarrays, and whole‐genome sequencing technology, provide fast, high‐throughput resistance gene detection [5]. However, their inability to detect unknown resistance genes and the fact that gene presence does not always correlate with phenotypic resistance limit their clinical utility [6]. In contrast, phenotypic methods directly assess bacterial activity under antibiotic pressure and provide a more accurate picture of resistance behavior. Among these, broth microdilution and disk diffusion (Kirby‐Bauer) are widely accepted as gold standard methods, as endorsed by the Clinical and Laboratory Standards Institute (CLSI) and the European Committee on Antimicrobial Susceptibility Testing (EUCAST) [5]. Nevertheless, as culture‐based approaches, these methods are inherently time‐consuming, typically requiring 18–24 h to yield results, and may take several weeks for slow‐growing pathogens such as *Mycobacterium tuberculosis* (*M. tuberculosis*) [7, 8]. Such delays may hinder timely treatment decisions, compromise therapeutic efficacy, and promote the spread of resistance. Consequently, there is an urgent need to develop rapid and reliable phenotypic methods to meet clinical demands for rapid AST.

**FIGURE 1:**
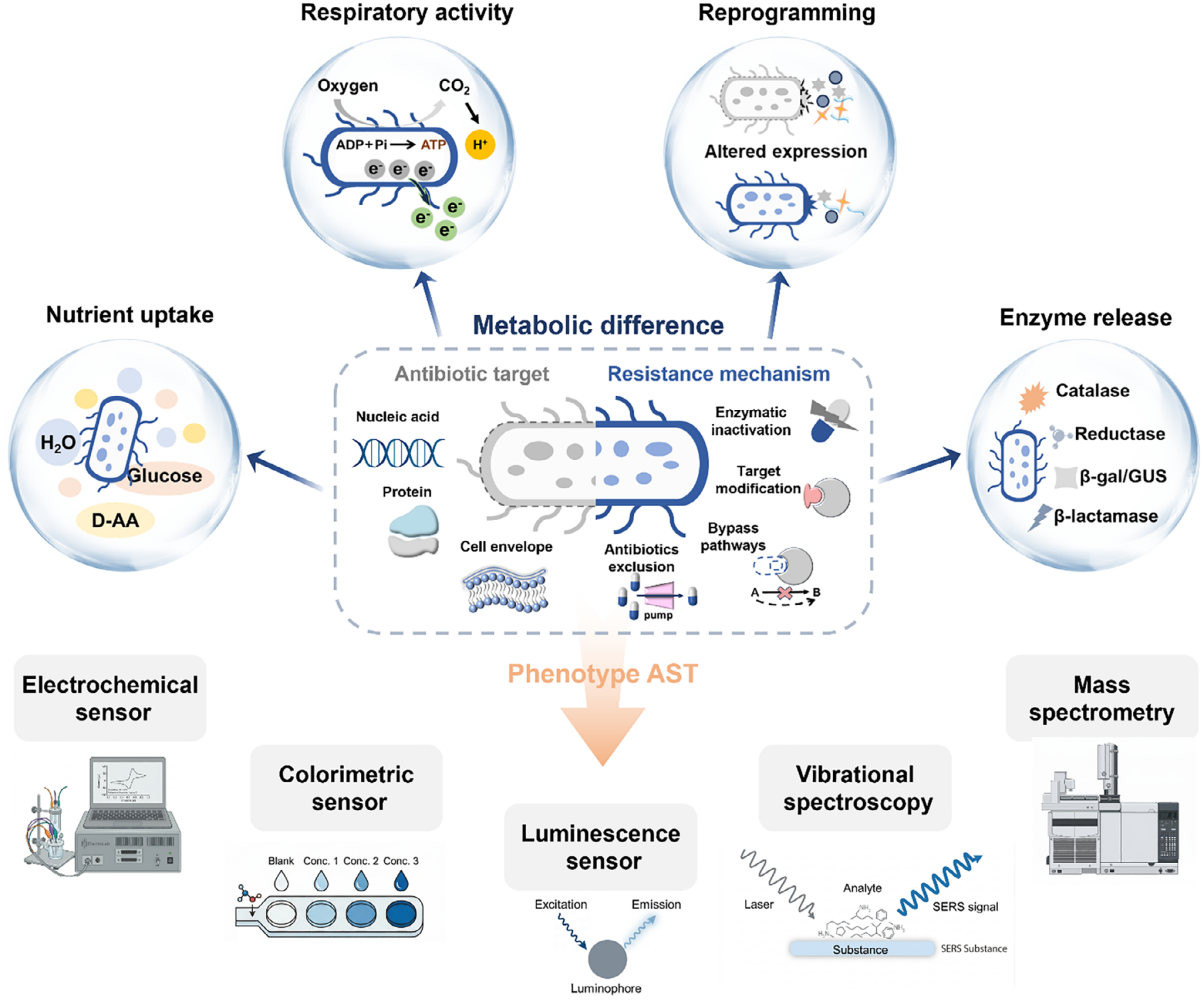
Schematic diagram illustrating metabolic biomarker‐based phenotypic AST to distinguish between susceptible and resistant strains.

Among emerging strategies, metabolism‐based AST has shown great promise, as the metabolic shifts of bacteria can occur within minutes to hours of antibiotic exposure [9, 10, 11], allowing the metabolism‐based AST to detect bactericidal activity early [12]. In recent years, numerous metabolism‐based AST have been developed, prompting investigations into various metabolic biomarkers for this purpose [13, 14, 15, 16, 17]. Despite these advances, comprehensive studies that systematically review these efforts are scarce. To address this gap, we summarize the major metabolic biomarkers and their associated detection strategies that have been applied to rapid phenotypic AST. These biomarkers are classified based on their biological relevance (Figure 1), including nutrient uptake, respiratory activity, metabolic reprogramming, and enzymatic function. We further compare these biomarker categories and their corresponding AST technologies in terms of their strengths, limitations, and diagnostic applicability, while also considering limitations in clinical settings. Meanwhile, this review has outlined the current understanding of how antibiotics influence bacterial metabolism, demonstrating the practical feasibility of using metabolism‐based biomarkers in AST.

## 2 Antibiotic Involved Mechanisms: Function and Resistance

Antibiotics exert their therapeutic effect by disrupting key physiological processes required for bacterial growth and survival [18]. Understanding how antibiotics perturb essential metabolism and how resistant bacteria compensate for these disruptions provides crucial insight into the biochemical basis of susceptibility. It also lays the foundation for identifying novel metabolic biomarkers capable of distinguishing susceptible and resistant phenotypes within minutes of antibiotic exposure [19, 20, 21].

### 2.1 Antibiotic Function

The bactericidal or bacteriostatic activity of antibiotics stems from the interference with three core biosynthetic systems: nucleic acid synthesis, protein synthesis, and cell envelope construction. As shown in Table 1, we have listed the antibiotic targets of major antibiotic classes. Disruption of these processes inevitably affects the physiological state of bacteria, thereby producing characteristic metabolic signatures of susceptibility [11, 22]. Notably, some antibiotics exert multiple inhibitory effects across these pathways.

**TABLE 1:** Major antibiotic classes, their molecular targets, metabolic consequences, and resistance mechanisms.

Antibiotic class	Target	Metabolic consequences	Resistance mechanisms	Ref.
Aminoglycosides	A‐site of 16S rRNA	Causing misreading and truncated proteins	Aminoglycoside‐modifying enzymes, 16S rRNA methyltransferases, reduced influx, or efflux pump expression	[30, 31, 49, 68]
Glycopeptides	D‐Ala–D‐Ala termini of lipid II PG precursors	Inhibiting the synthesis of peptidoglycan to disrupt cell membrane synthesis	Intrinsic resistance in Gram‐negative bacteria, peptidoglycan precursors, modifying or hydrolyzing enzymes	[41, 69]
Macrolides	23S rRNA	Truncating peptide chains to inhibit protein synthesis	23S rRNA methylation, efflux pumps, or ribosomal protein mutations	[34, 70]
Nitroimidazoles/Nitrofurans	DNA bases	Breaking DNA strands	Loss‐of‐function mutations in activating nitroreductases, decreasing anaerobic respiration enzymes, or efflux pump upregulation	[24, 71, 72, 73]
Oxazolidinones	50S ribosomal subunit	Blocking translation initiation	Methyltransferase, ribosomal protein mutations	[35, 74]
Quinolones	DNA gyrase and topoisomerase IV	Inhibiting DNA replication and decatenation	Target mutations, protection proteins, enhanced efflux, or decreased porin permeability	[55, 69, 75, 76]
Rifamycins	β‐subunit of RNA polymerase (rpoB)	Blocking transcription elongation and halting protein synthesis	rpoB mutation, rifamycin‐modifying enzymes, or reduced permeability	[77, 78]
Sulfonamides	Dihydropteroate synthase at the p‐aminobenzoic acid ‐binding site	Blocking THF regeneration, impairing one‐carbon metabolism, stalling DNA replication, and RNA synthesis	Dihydropteroate synthase mutations, porin loss, or efflux pumps	[79]
Tetracyclines	16S rRNA at the aminoacyl‐tRNA binding site of the 30S subunit	Blocking aminoacyl‐tRNA entry to decrease protein synthesis	Ribosomal protection proteins, efflux pumps, or enzymatic inactivation	[80, 81, 82]
Trimethoprim	Dihydrofolate reductase (DHFR) catalytic active site	Blocking THF regeneration, impairing one‐carbon metabolism, stalling DNA replication	DHFR isoforms, DHFR mutations, or metabolic bypass via increased folate pathway flux	[83, 84, 85]
β‐Lactams	PBPs	Inhibiting PG cross‐linking, weakening the cell wall, and leakage of metabolites	β‐lactamases, PBP mutations, acquisition of PBP2a, porin loss, or efflux pumps	[39, 40, 86, 87]

Antibiotics that target nucleic acid synthesis act through three primary mechanisms: (i) inhibiting tetrahydrofolate (THF) synthesis, an essential one‐carbon carrier required for nucleotide biosynthesis [23]; (ii) inhibiting DNA replication, a semi‐conservative process involving the unwinding of the double helix by DNA gyrase, followed by strand elongation via DNA polymerase [24, 25]; (iii) inhibiting RNA synthesis, which depends on RNA polymerase to transcribe DNA or RNA templates [26]. These interruptions disrupt nucleic acid synthesis and deplete nucleotide and cofactor pools, collapse adenosine triphosphate (ATP) production, and trigger oxidative stress through excess NADH oxidation, ultimately causing bacterial death [27, 28, 29].

For protein synthesis inhibition, antibiotics are also able to target the bacterial ribosome, a complex macromolecular machine responsible for translating genetic information into proteins, which consists of two subunits, the 30S and 50S. The antibiotics can target the recognition site of messenger RNA and the binding site of transfer RNA (tRNA) on the 30S subunit, causing codon misreading and the accumulation of misfolded proteins [30, 31, 32, 33]. Meanwhile, some antibiotics target the 50S subunit, including binding the peptidyl transferase center, obstructing the nascent peptide tunnel to prevent translocation of peptidyl‐tRNA [34] or directly binding to the 50S ribosomal subunit to prevent the formation of the 70S initiation complex, effectively preventing the assembly of a functional ribosome [35]. These disruptions of translation accumulate misfolded proteins and activate stress‐response pathways that consume large amounts of energy while elevating reactive oxygen species (ROS), ultimately causing bacterial death or dormancy [36].

The bacterial cell envelope, comprising the cell wall and membrane, is essential for structural integrity and homeostasis. Most antibiotics interfere with peptidoglycan (PG) biosynthesis, the primary component of the cell wall [37, 38]. They can bind to penicillin‐binding proteins (PBPs), blocking the transpeptidation and PG cross‐linking [39, 40] or attach to PG precursors, preventing polymerization [41]. Other antibiotics directly disrupt the cell membrane, mainly composed of lipids, which also serves as a scaffold for cellular metabolism and protein regulation [42]. For example, Polymyxins interact with lipopolysaccharides of Gram‐negative bacteria, displacing divalent cations and destabilizing the membrane [43, 44]. Daptomycin inserts into the cytoplasmic membrane of Gram‐positive bacteria in a calcium‐dependent manner, forming ion‐permeable channels that cause depolarization and ion efflux [45]. These antibiotics destroy the barrier function of the cell envelope, causing uncontrolled leakage of cytoplasmic metabolites, including ATP, ions, and other small molecules, into the extracellular space [46].

### 2.2 Resistance Mechanisms

While antibiotics target essential bacterial processes, resistant bacteria evolve strategies that neutralize or evade their effects. We have listed the most resistant mechanisms of major antibiotic classes in Table 1. The resistance mechanisms can be categorized into enzymatic inactivation, target modification, bypass pathways, and antibiotic exclusion [47, 48]. Antibiotic inactivation is the major mechanism of antibiotic resistance. Many bacteria produce enzymes that chemically modify or degrade antibiotics before they reach their targets [49, 50, 51, 52]. For example, β‐lactamases hydrolyze the β‐lactam ring, rendering β‐lactam antibiotics inactive [19, 53]. Target modification reduces or prevents drug binding without compromising essential cellular function [54]. For example, methylation of 23S or 16S rRNA by specific methyltransferases confers resistance to macrolides and aminoglycosides [55]. Bypass pathways are a strategy designed to circumvent antibiotics by creating an alternative route, rendering the original target site redundant. For example, β‐lactams‐resistant bacteria express PBP2a to form PG, an alternative functional enzyme with low affinity for β‐lactams [56]. Antibiotic exclusion is another major resistance strategy for limiting intracellular antibiotic accumulation. Loss or mutation of outer membrane porins restricts antibiotic entry, as observed with OmpK36 in *Klebsiella pneumoniae* (*K. pneumoniae*) [57] and OmpC in *Escherichia coli* (*E. coli*) [58]. Concurrently, energy‐dependent efflux pumps actively expel antibiotics from the cell [59], existing in most antibiotic‐resistant bacteria [60].

Each strategy not only protects bacterial structures or functions but also imposes distinct metabolic costs that reshape cellular physiology [61]. In these strategies, many resistant strains exhibit metabolic adaptation for survival under antibiotic stress. Rather than simply slowing growth, these bacteria actively alter central metabolic pathways to maintain cellular homeostasis [10, 62, 63]. For example, in some resistant bacteria, pyruvate flux is redirected from oxidative phosphorylation toward fermentative pathways to reduce electron transport chain activity and minimize ROS generation [64]. At the same time, amino acid metabolism is upregulated to support the biosynthesis of resistance‐related proteins, including efflux pumps, antibiotic‐modifying enzymes, and structural proteins of the cell envelope [65, 66]. These metabolic shifts are tightly coupled with transcriptional regulation, resulting in distinct RNA expression profiles compared with susceptible strains [67]. Collectively, these resistance strategies not only protect essential cellular processes but also impose characteristic metabolic shifts, laying the foundation for metabolism‐based biomarkers for rapid phenotypic antibiotic susceptibility testing.

## 3 Metabolic Biomarkers in Emerging AST

In the previous section, we discussed how bacterial metabolism may change under antibiotic pressure. Here, we summarize emerging metabolism‐based biomarkers used for rapid AST. For clarity, these biomarkers are grouped based on the main metabolic processes affected by antibiotic exposure: nutrient uptake, respiratory activity, metabolic reprogramming, and enzymatic function.

### 3.1 Nutrient Uptake‐Based Biomarker

The uptake of nutrients is closely linked to bacterial growth, proliferation, and energy balance. Accordingly, tracking the uptake and use of key nutrients can provide a readout of bacterial physiological status without prior species identification, although it generally offers limited insight into the resistance mechanisms or the specific mode of antibiotic action. Among various options, glucose, H_2_O, and D‐amino acids (D‐AA) are especially important because of their key roles in bacterial metabolism and the availability of established detection methods for their analysis.

#### 3.1.1 Glucose or H_2_O Uptake

**FIGURE 2:**
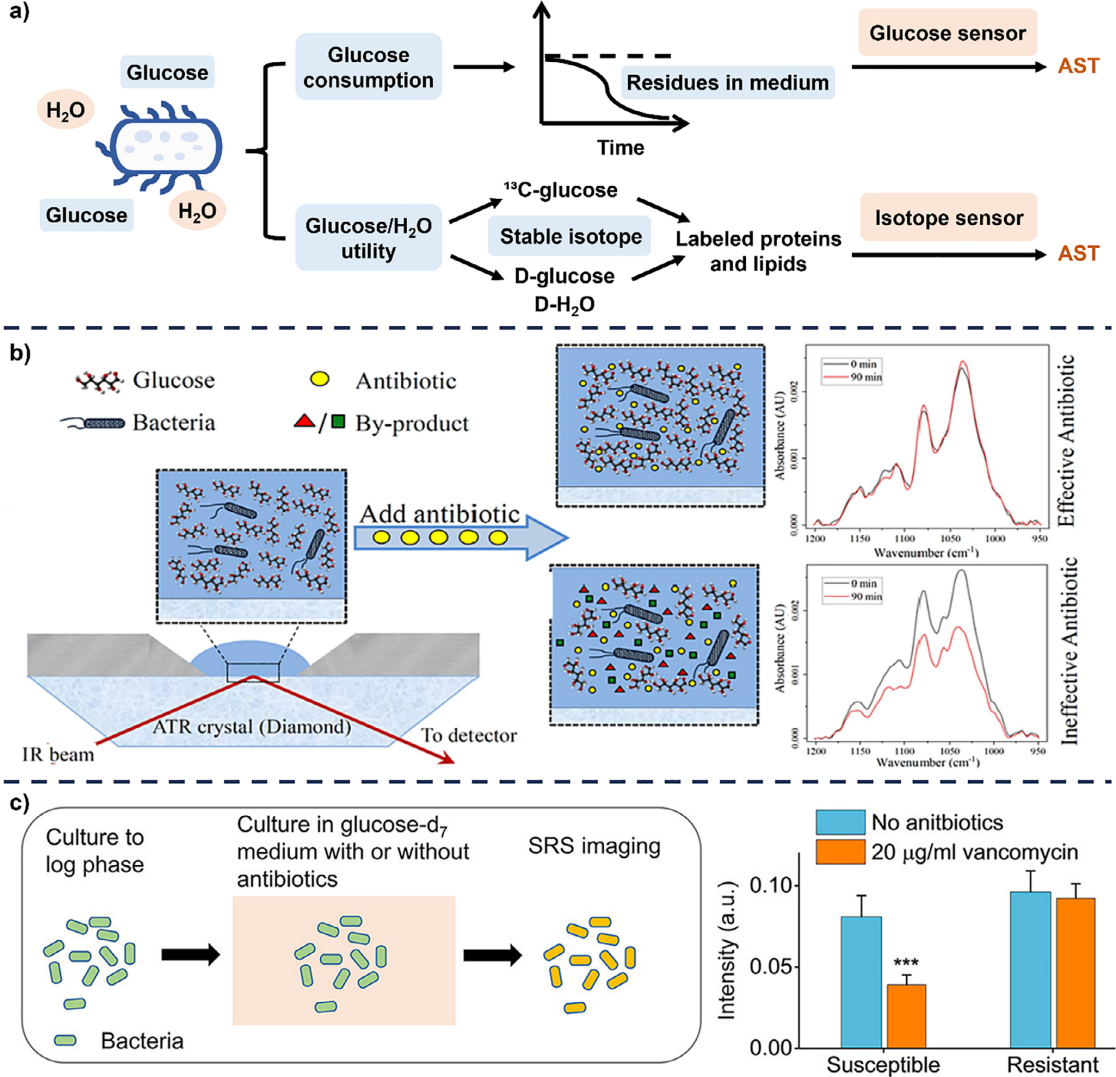
AST platforms via monitoring glucose and H_2_O uptake. (a) Overview of AST strategies that track glucose and water uptake, including measuring glucose consumption with glucose sensors and directly tracing glucose and H_2_O utilization via isotope labeling sensors. (b) Principle of an IR‐ATR assay developed by Ederth and co‐workers et al. for detecting residual glucose in the culture medium. The method utilized characteristic carbohydrate absorption bands in the 1200–950 cm^−1^ range. In the upper graph, unchanged carbohydrate levels indicated an effective antibiotic dose, while in the lower graph, reduced absorption signals indicate continued bacterial metabolism, reflecting antibiotic resistance. Reproduced with permission [91], under CC BY license. (c) Principle of a glucose utilization sensor using SRS, developed by Cheng and co‐workers et al. The analysis included a statistical comparison of average C–D bond signal intensities in bacterial cells, with and without treatment using 20 µg mL^−1^ vancomycin, providing a rapid AST within 0.5 h. Reproduced with permission [94], Copyright 2018 American Chemical Society.

Glucose, a key carbon source, is essential for energy generation and biosynthesis in many microorganisms. Due to its rapid kinetics and ease of quantification [88], bacterial metabolic activity can be rapidly assessed by monitoring their glucose consumption rates and intracellular glucose utilization, enabling rapid AST (Figure 2a). The consumption of glucose is typically measured by detecting the residues in the medium, with detection techniques including electrochemical methods, conventional analytical methods, and spectroscopic methods [89, 90]. As shown in Figure 2b, Ederth and co‐workers et al. [91] used infrared attenuated total reflection (IR‐ATR) spectroscopy to monitor glucose consumption immediately after 24 h of dosing with antibiotics. They assessed bacterial activity by analyzing changes of carbohydrate molecular vibrations (1200–900 cm^−1^) and the emergence of carboxyl group's absorption bands at 1561 cm^−^^1^ (asymmetric stretching) and 1416 cm^−1^ (symmetric stretching). The rate of this decline was subsequently used to identify bacterial resistance. Similarly, Neumaier and co‐workers et al. [89] employed a liquid chromatography‐mass spectrometry (LC‐MS) to monitor the residual glucose in the media, assessing bacterial metabolic activity under antibiotic stress. Glucose consumption can also be measured on other clinical chemistry analyzers, enabling the integration of AST into routine laboratory workflows.

In addition to measuring nutrient consumption, directly assessing their utilization provides a more intuitive approach. Given that glucose and water are essential elements for the biosynthesis of macromolecules such as lipids and proteins, isotopic labeling of these compounds provides a valuable approach for tracking their utilization [92]. Cheng and co‐workers et al. [93] employed ^13^C‐labeled glucose and quantified protein synthesis efficiency by analyzing the ^13^C‐protein replacement ratio using dual‐wavenumber mid‐infrared photothermal imaging. They demonstrated that the ^13^C‐protein replacement ratio served as a reliable metabolic biomarker for determining *E. coli* susceptibility to various antibiotics with just 1 h of treatment. They further extended the method by supplementing the culture medium with deuterium‐labeled glucose (glucose‐d_7_) and heavy water (D_2_O), respectively, which were incorporated into bacterial lipids and proteins to form characteristic C–D bonds, enabling rapid metabolic‐based AST [94, 95]. As shown in Figure 2c, when bacteria were cultured in glucose‐d7‐containing medium with or without antibiotics, resistant strains metabolically incorporated deuterium, forming C‐D bonds in vivo. These characteristic bonds were detected by stimulated Raman scattering (SRS), revealing distinct metabolic differences between susceptible and resistant strains within just 0.5 h of antibiotic exposure, as indicated by significant variations in signal intensity [94]. Similarly, Lee and co‐workers et al. [96] used matrix‐assisted laser desorption/ionization mass spectrometry (MALDI‐MS) to monitor bacterial growth by detecting the deuterium incorporation into lipids.

#### 3.1.2 D‐Amino Acids Incorporation

**FIGURE 3:**
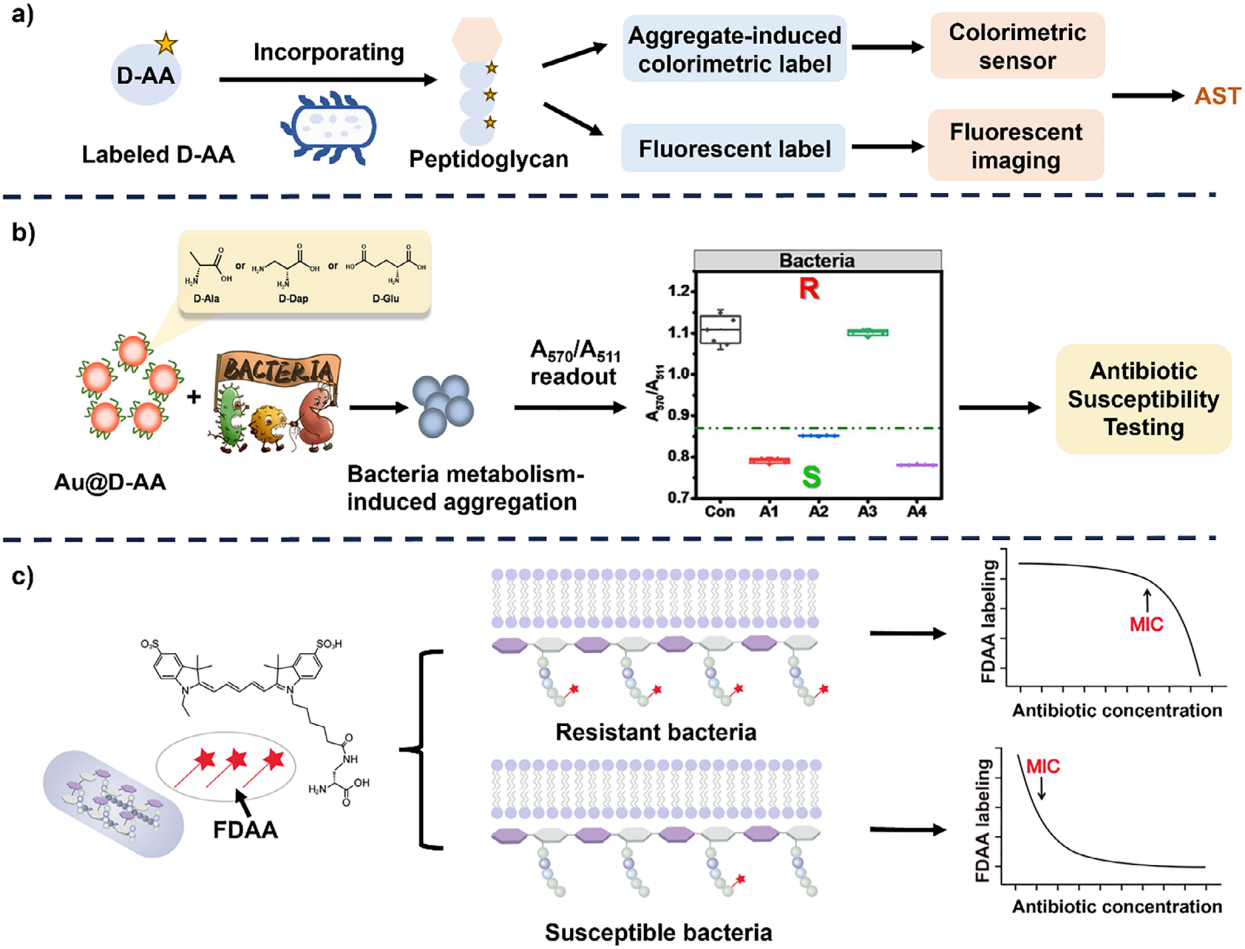
AST platforms via monitoring D‐AA incorporation. (a) Overview of AST strategies using D‐AA incorporation, including aggregation‐induced colorimetric probes and fluorescently labeled D‐AAs, along with their detection platforms. (b) Principle of a colorimetric sensing platform developed by Liu and co‐workers et al., based on bacterial metabolism‐driven aggregation of D‐AA probes. After incubation with bacteria and antibiotics, resistant strains promoted probe aggregation and caused visible color changes, while susceptible strains did not, allowing for rapid visual AST. Adapted with permission [97], Copyright 2018 American Chemical Society. (c) Principle of a fluorescence‐based AST approach using FDAAs, developed by Wang and co‐workers et al. Bacteria were incubated with FDAA and antibiotics for 4 h, then analyzed by flow cytometry for FDAA labeling. Resistant strains maintained higher FDAA labeling intensity until exposed to high antibiotic doses, while susceptible strains showed reduced labeling even at low drug levels. MICs were calculated from the FDAA labeling curves. Adapted with permission [101], under CC BY license.

D‐AAs are essential components of bacterial peptidoglycan, which plays a crucial role in maintaining the structural integrity and rigidity of the cell wall [97, 98]. During bacterial growth, the incorporation of exogenous D‐AAs into the peptidoglycan layer reflects active cell wall biosynthesis and can therefore serve as a useful biomarker for AST [99, 100]. Capitalizing on this incorporation mechanism, researchers have developed aggregation‐induced colorimetric sensors and fluorescence imaging strategies to monitor bacterial metabolic activity dynamically (Figure 3a). Liu and co‐workers et al. [97] developed a colorimetric sensor array by functionalizing gold nanoparticles (AuNPs) with three types of D‐AAs (Figure 3b). The metabolic incorporation of these D‐AAs into peptidoglycan triggered aggregation of AuNPs, resulting in visible color changes. By calculating colorimetric signal changes, the antibiotic resistance was discriminated against. Meanwhile, different bacteria exhibited different metabolic abilities toward different D‐AA and diverse AuNPs aggregation. Therefore, the unique colorimetric responses enabled the identification of bacterial species. In addition, fluorescent‐labeled D‐AA can serve as a probe to observe the synthesis in bacterial cell walls, producing measurable fluorescence signals. Building on the concept, Wang and co‐workers et al. [100, 101] synthesized fluorescent‐labeled D‐AAs (FDAAs) by conjugating D‐AAs with fluorophores. As shown in Figure 3c, when bacteria were co‐incubated with FDAAs and antibiotics, resistant strains incorporated significantly more FDAAs into their cell walls compared to susceptible strains. Fluorescence intensity was quantified via flow cytometry, and the minimum inhibitory concentration (MIC) was determined by analyzing intensity trends across varying antibiotic concentrations in a few hours.

### 3.2 Respiratory Activity‐Derived Biomarkers

Bacterial respiration is the primary pathway for energy generation, reflecting the metabolic activity of the cell [102]. During aerobic or anaerobic respiration, electrons are transferred through the electron transport chain to terminal acceptors, driving ATP synthesis and producing metabolites such as CO_2_ and organic acids to alter pH. These measurable changes serve as rapid physiological biomarkers for bacterial viability, providing a basis for AST.

#### 3.2.1 pH Variation

**FIGURE 4:**
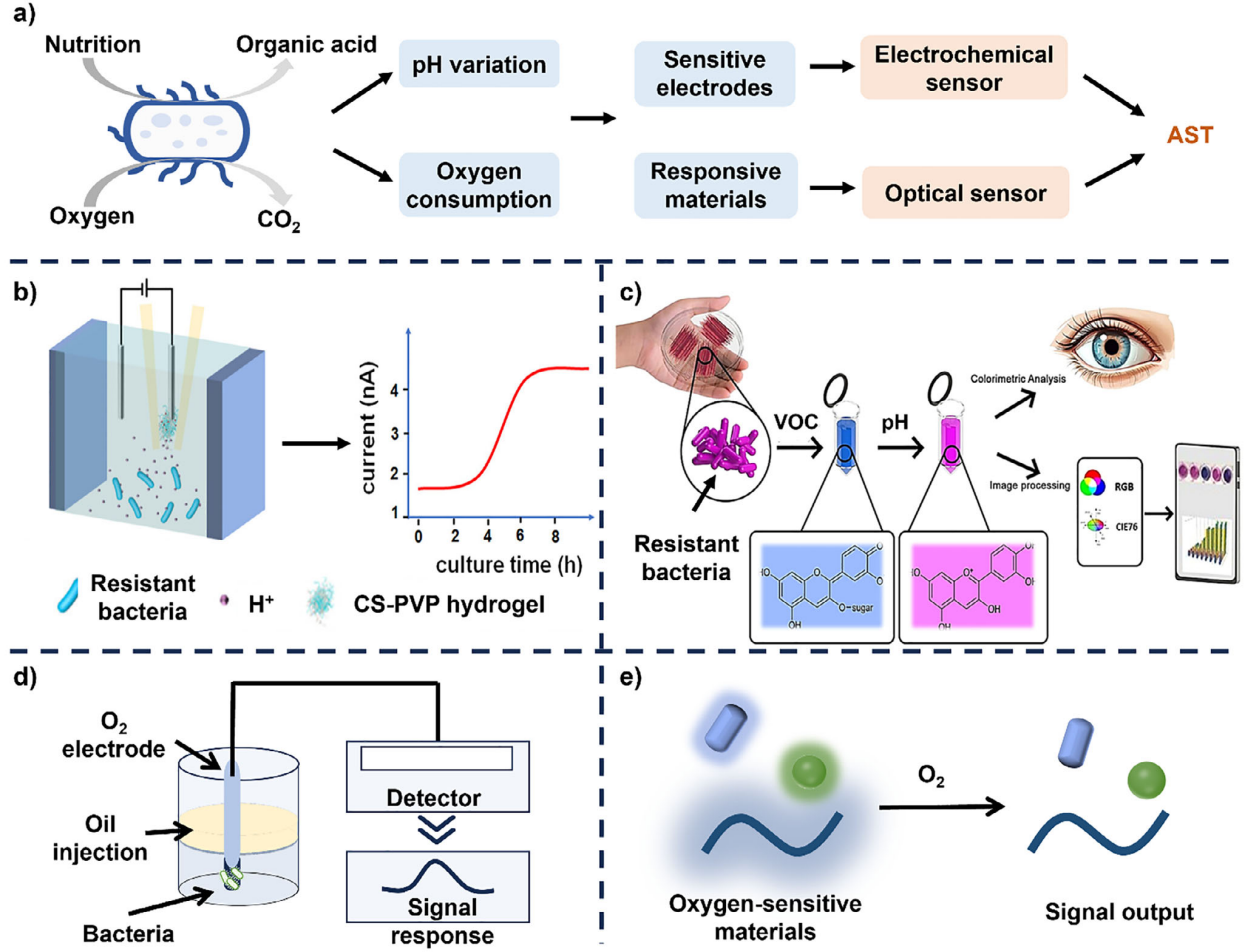
AST platforms via monitoring pH variation and oxygen consumption in the culture medium. (a) Overview of AST utilizing pH variations and oxygen consumption as indicators for AST. (b) Principle of an electrochemical sensor developed by Wu and co‐workers et al., employing CS‐PVP hydrogel as a pH‐sensitive electrode. Antibiotic‐resistant bacteria maintain metabolic activity after incubation with antibiotics, resulting in a pH decrease, transducing an electrochemical signal for AST readout. Adapted with permission [104], Copyright 2024, American Chemical Society. (c) Principle of a pH‐responsive material‐based sensor developed by Ocsoy and co‐workers et al. As the pH of the medium becomes lower, the color visibly changes from blue to pink, allowing visual identification of bacterial resistance. Reproduced with permission [109], Copyright 2025, American Chemical Society. (d) Principle of an electrochemical sensing platform using oxygen‐sensitive electrodes for real‐time monitoring of dissolved oxygen levels. Bacteria immobilized on the electrode surface consume oxygen during metabolism, resulting in measurable electrochemical signal changes that distinguish resistance from susceptible strains. (e) Principle of a sensor utilizing oxygen‐sensitive materials. These materials measure oxygen consumption through changes in emission signals, enabling rapid AST.

During respiration, bacteria release CO_2_, which reduces the pH through carbonic acid formation in the medium. Additionally, they metabolize nutrients (e.g., sugars) into organic acids through fermentation, further acidifying the culture medium (Figure 4a). Therefore, pH variations serve as indirect biomarkers of bacterial metabolic activity and have been increasingly explored as sensitive signals for rapid AST [15, 103, 104]. A range of pH‐based sensors has thus emerged for AST, falling broadly into two categories: electrochemical and colorimetric methods. Electrochemical methods often utilize pH‐sensitive electrodes, including metal oxide electrodes [105], conductive polymers [106], and carbon nanomaterial [107], among others. These electrodes respond to environmental changes in H^+^ concentration through mechanisms such as protonation/deprotonation, redox reactions, or charge transfer, enabling real‐time monitoring of pH fluctuations associated with bacterial metabolic activity. Wu and co‐workers et al. [104] developed a novel pH electrode based on chitosan/polyvinylpyrrolidone (CS‐PVP) hydrogel‐filled nanocapillaries (Figure 4b). In this system, a decrease in pH induced the amino groups (─NH_2_) of chitosan to bind protons (H^+^), forming protonated groups (─NH_3_^+^) and thereby significantly enhancing the hydrogel's hydrophilicity. This protonation led to an expansion of the nanocapillary pore size and an increase in ionic current intensity, which were effectively detected in real time for evaluating *E. coli* susceptibility. Notably, a decrease in pH also increases the conductivity of the medium, enabling an impedance‐based sensor for AST [108]. Colorimetric pH detection is an optical strategy that relies on pH‐responsive materials, including natural pigments [109], synthetic chemical biomarkers [110], or covalent organic frameworks [111]. Ocsoy and co‐workers et al. [109] employed an optical, colorimetric approach based on the pH sensitivity of anthocyanins, a class of natural pigments for AST. As shown in Figure 4c, in the presence of antibiotics, the metabolism of resistant bacteria lowered the pH of the microenvironment to trigger anthocyanin protonation, causing the solution color to change from blue to pink, while susceptible bacteria cannot make the color change. This technology allowed for visual readout and could be combined with smartphone image analysis for semi‐quantitative assessment. Together, these approaches demonstrate that pH is not merely a passive environmental parameter but an active metabolic readout for rapid AST.

#### 3.2.2 Oxygen Consumption

Oxygen is essential for bacterial respiratory metabolism, particularly in aerobic bacteria, whose growth and activity depend on oxygen uptake (Figure 4a) [112]. Therefore, developing sensors that monitor oxygen consumption provides an efficient strategy for evaluating bacterial responses to antibiotics, especially for slow‐growing bacteria. Currently, oxygen concentrations are commonly monitored in real time using electrochemical methods or oxygen‐sensitive nanomaterial‐based sensors [113, 114, 115]. Electrochemical methods typically involve immobilizing bacteria on electrode surfaces (Figure 4d). Zhang et al. [116] established an electrochemical method by attaching *M. tuberculosis* to the surface of an oxygen electrode. This method utilized glycerol injection to create an airtight seal, which effectively monitored oxygen consumption by generating an electric signal under antibiotic stress, thereby reducing the AST time from 11 to 4 days.

Oxygen‐sensitive nanomaterials‐based sensors typically incorporate phosphorescent or fluorescent dyes, whose emission intensity is quenched in the presence of oxygen via dynamic quenching. As bacteria respire, they consume dissolved oxygen in the surrounding medium, resulting in a measurable increase in the emission signal (Figure 4e). Dittrich and co‐workers et al. [115] used oxygen‐sensitive nanoprobes to develop a microfluidic device, enabling AST within 2–3 h. In this platform, bacterial metabolic activity would affect oxygen levels, altering the fluorescence signal intensity to indicate antibiotic susceptibility or resistance. Chung and co‐workers et al. [117], Nagl and co‐workers et al. [118] and Gijs and co‐workers et al. [114] also used a similar principle to design sensors to conduct AST within a few hours.

The above‐mentioned approaches primarily assess dissolved oxygen levels in the suspension medium. However, Cate and co‐workers et al. [119] directly measured the oxygen concentration and consumption on the cell membrane. They embedded oxygen‐sensitive polymer nanosensors into the cell membrane of *Pseudomonas aeruginosa* (*P. aeruginosa*). By measuring the response of the nanosensors under antibiotic treatment, they gained insights into biofilm behavior and obtained information on the MIC and time‐killing kinetic curves. Notably, the oxygen sensor significantly improved the AST detection time, but it is only applicable to aerobic bacteria and facultative anaerobic bacteria.

#### 3.2.3 Extracellular Electron Transfer

**FIGURE 5:**
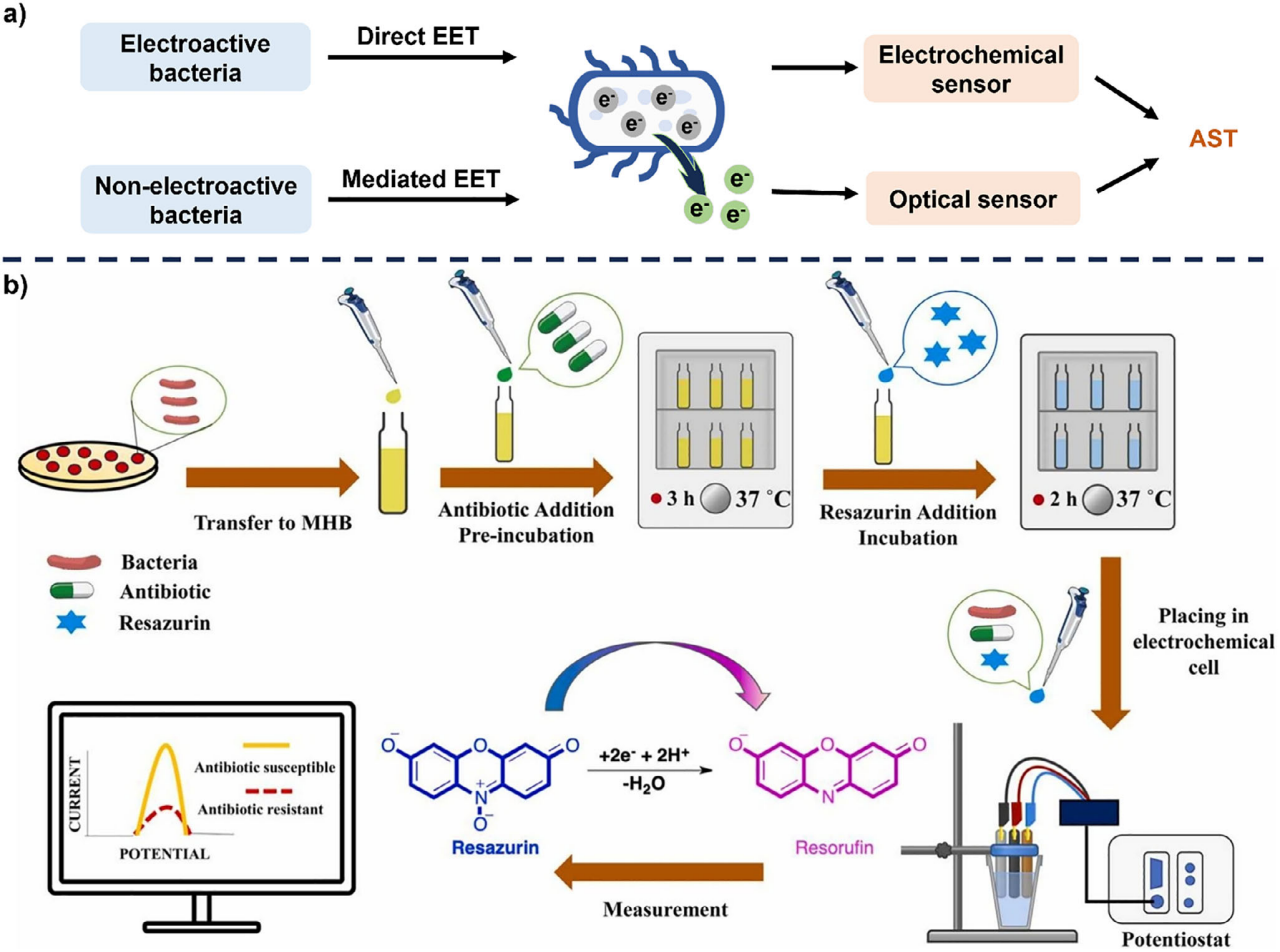
AST platforms via detecting extracellular electrons. (a) Overview of AST strategies utilizing EET as an indicator, including the pathway of extracellular electron transfer and detection platforms based on electron‐responsive properties. (b) Principle of an electrochemical sensor developed by Topkaya and co‐workers et al., employing a resazurin‐modified electrode for signal output. Metabolically active bacteria transfer electrons to reduce resazurin to resorufin, which causes a decrease in the reduction current and allows for distinguishing resistant from susceptible strains. Reproduced with permission [125], Copyright 2025 Elsevier B.V.

In bacterial respiration, the electron transport chain plays a central role by shuttling electrons derived from metabolic substrates, resulting in a substantial electron flux. In electroactive bacteria, these electrons can be transferred to external acceptors through extracellular electron transfer (EET) mechanisms (Figure 5a). Upon delivery of electrons to an electrode surface, measurable bioelectrical currents are produced. The amplitude of these currents reflects bacterial metabolic activity, allowing AST to be performed via voltage or current measurements [12, 14]. Building on this principle, Choi and co‐workers et al. [120] designed an 8‐channel paper‐based AST platform to harvest bacterial electrons. Voltage changes were monitored over 900 s to determine bacterial resistance, whose results closely matched those of the conventional broth microdilution method. However, this approach is limited to inherently electroactive bacteria [121], such as *P. aeruginosa*, which utilizes endogenous redox‐active appendages for EET [122, 123].

Exogenous redox compounds can be employed to address this limitation, including ferricyanide [124], resazurin [125, 126], and phenazine methosulfate [121]. They function as electron shuttles through reversible redox reactions, facilitating electron transfer between bacteria and electrodes, thereby enabling mediated EET in non‐electroactive strains. Recently, Topkaya and co‐workers et al. [125] engineered a resazurin‐based electrochemical sensor for detecting antibiotic resistance in *Stenotrophomonas maltophilia (S. maltophilia)* and *Shigella sonnei (S. sonnei)* within 6 h. As shown in Figure 5b, after pre‐incubation with antibiotics, metabolically active bacteria can reduce resazurin to resorufin, thereby lowering the reduction current. In addition to generating electrical signals, exogenous redox media often produce color changes, which are used for visual reading of AST. For example, potassium ferrocyanide is reduced by extracellular electrons to ferrocyanide, changing color from yellow to colorless. Resazurin is reduced to resorufin, changing color from blue to pink [127]. Biswas and co‐workers et al. [128] created a paper‐strip‐embedded masking tape system via resazurin redox colorimetry. In this system, after 4 h of bacterial culture and 10 h of antibiotic exposure, the resazurin underwent a reduction reaction, changing color from blue to pink, indicating metabolic activity. While colorimetric readings require longer incubation times to accumulate sufficient electrons, their visual interpretation advantage, without the need for instruments, makes them suitable for point‐of‐care testing applications [129].

#### 3.2.4 ATP Level

**FIGURE 6:**
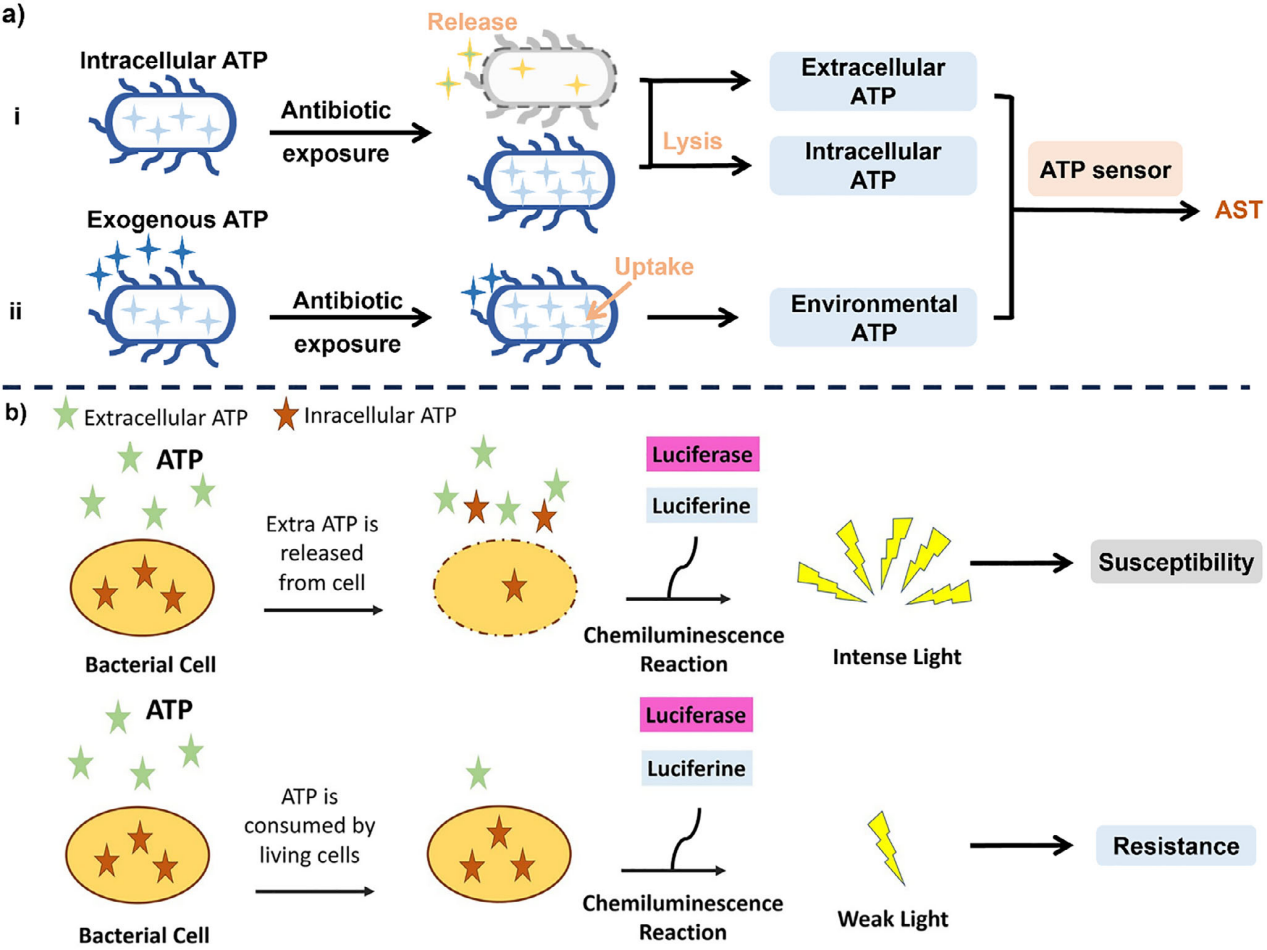
AST platforms via monitoring ATP levels. (a) Principle of an ATP sensor‐based AST strategy. (i) Principle of an ATP sensor‐based AST by monitoring extracellular ATP concentrations after antibiotic exposure and intracellular ATP levels following bacterial lysis. In susceptible strains, membrane integrity is compromised, stopping ATP production and causing ATP to leak out, which results in elevated extracellular ATP but decreased intracellular ATP. In resistant strains, ATP remains inside the cell and continues to be produced, leading to higher total ATP without a significant increase in extracellular ATP. (ii) Principle of an ATP sensor‐based AST that uses exogenously supplied ATP without bacterial lysis. After antibiotic exposure, resistant strains actively consume the exogenous ATP, leading to a measurable decrease in environmental ATP levels. In contrast, susceptible strains cannot utilize the exogenous ATP and may even release intracellular ATP, causing an increase in environmental ATP. (b) The principle of bioluminescent ATP sensor‐based AST by Kocagoz and co‐workers et al. The susceptible bacteria do not use ATP in the medium with antibiotics and leak intracellular ATP outside, resulting in light emission. If bacteria are resistant, they grow and consume ATP, preventing light production. Reproduced with permission [139], under CC BY license.

ATP is the universal energy currency in bacteria and a direct product of cellular respiration. Under antibiotic stress, susceptible bacteria exhibit membrane dysfunction that allows passive ATP leakage and reduced ATP levels, whereas resistant strains tend to maintain ATP production in the cell [130]. Therefore, comparing ATP levels in the extracellular medium or bacterial cells after antibiotic exposure has become a practical approach for AST (Figure 6a). Several methods are available for ATP quantification, including biochemical luminescence assays [131, 132], aptamer‐based sensors [133, 134], and metal organic framework ‐based fluorescent sensors [135, 136]. Among these, luciferin‐based bioluminescence is the most widely used, and most commercial ATP assay kits are based on this principle. The bioluminescence assay involves the oxidation of D‐luciferin catalyzed by luciferase in the presence of Mg^2+^, O_2_, and ATP, producing light that is directly proportional to ATP concentration and can be detected with high sensitivity using a luminometer [137]. Spitz and co‐workers et al. [138] employed a thermostable luciferase–luciferin system to monitor extracellular ATP in real‐time, correlating luminescence intensity with cell density to estimate viable cell counts. For active cells, extracellular ATP concentrations are significantly lower than intracellular levels (1–5 mM), typically accounting for only 3%–5% of total ATP in cultures. Due to limitations in signal sensitivity, bacteria lysis‐based assays for the detection of intracellular ATP remain the dominant approach in rapid AST platforms.

To address the need of cell lysis, Kocagoz and co‐workers et al. [139] have been developed an indirect strategies. They introduced exogenous ATP (20 mM) into bacterial cultures during antibiotic exposure. After incubation, a luciferin‐luciferase reaction mixture was added. In susceptible strains, cell death and membrane damage resulted in ATP accumulation in the medium, leading to strong bioluminescence. In contrast, resistant bacteria consumed ATP during growth, producing little or no light (Figure 6b). Thus, light intensity served as an indirect biomarker of bacterial growth and antibiotic susceptibility. However, although ATP is a reliable biomarker for AST, different bacterial species exhibit varying rates of ATP consumption. This makes it challenging to optimize the ATP concentration in the medium, as it is difficult to establish a universal level that works equally well for all species.

### 3.3 Metabolism Reprogramming‐Based Biomarker

Metabolic reprogramming is one of the initial responses by which bacteria adapt to environmental stress. Upon antibiotic exposure, susceptible bacteria rapidly reorganize their metabolic pathways, leading to transcriptional reconfiguration and transient attempts to restore homeostasis. However, after prolonged exposure, typically within a few hours, susceptible strains undergo metabolic collapse characterized by disrupted energy production and biosynthetic pathways. In contrast, resistant strains maintain metabolic activity, often through protective mechanisms such as efflux pumps, target modification, or enhanced repair systems. These distinct metabolic adjustments are reflected not only at the transcriptional level but also in metabolite profiles, providing early indicators of antimicrobial susceptibility before growth inhibition becomes evident.

#### 3.3.1 Differentially Expressed RNA

**FIGURE 7:**
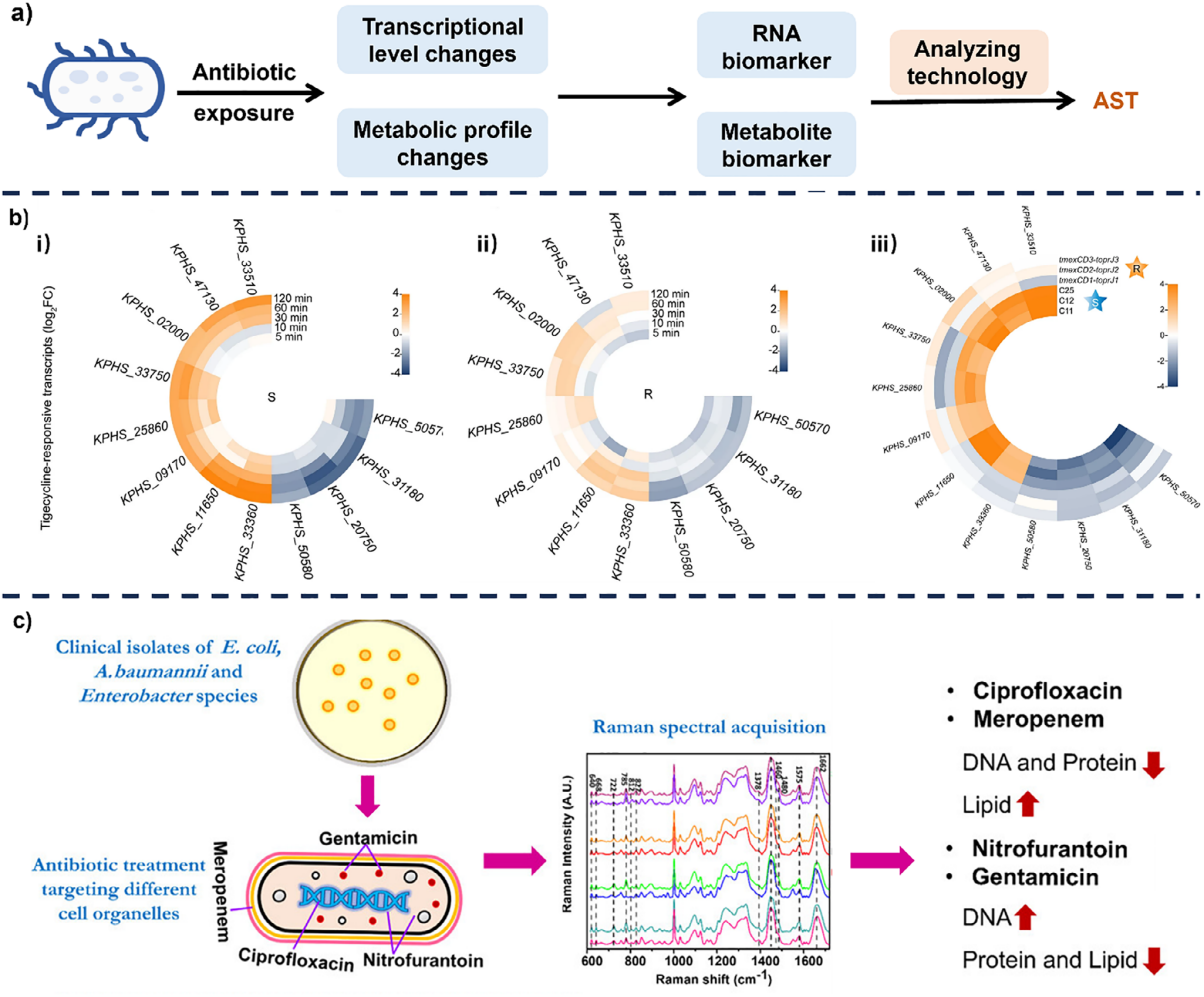
AST platforms via detecting differentially expressed RNA or metabolites. (a) An AST strategy based on transcriptional level and metabolite profile alterations. Transcriptomic analysis is conducted to compare bacterial gene expression profiles before and after antibiotic exposure, identifying differentially expressed RNA biomarkers. With antibiotic exposure, metabolomic profiling is performed to compare resistant and susceptible strains, identifying differentially expressed metabolites as biomarkers. These biomarkers are subsequently measured using analytical techniques to enable rapid AST. (b) Results of RNA‐based AST developed by Liu and co‐workers et al. upon tigecycline exposure in *K. pneumoniae*. (i, ii) Heatmaps showing the expression dynamics of 12 RNA biomarkers across different tigecycline exposure durations. (iii) Validation heatmap of the 12 biomarkers in tmexCD‐toprJ‐negative (denoted as S) and various tmexCD‐toprJ‐positive tigecycline strains (denoted as R) after tigecycline exposure. Reproduced with permission [142], under CC BY license. (c) AST platform by Umapathy and co‐workers et al. using SERS to detect metabolic changes. Ciprofloxacin‐ and meropenem‐treated susceptible strains showed decreased Raman intensities for DNA and proteins, with increased lipid signals. Gentamicin‐ and nitrofurantoin‐treated susceptible strains exhibited increased nucleic acid bands and decreased protein and lipid bands. Reproduced with permission [149], Copyright 2023, American Chemical Society.

RNA, as the fastest and most dynamic molecular biomarker, reflects metabolic changes rapidly. Within minutes of antibiotic exposure, susceptible bacteria upregulate metabolism‐related RNAs, whereas resistant strains exhibit minimal transcriptional changes. Therefore, by comparing the transcriptome profiles before and after antibiotic exposure, differentially expressed RNA can serve as a biomarker to rapidly identify bacterial susceptibility (Figure 7a) [140]. Notably, each strain has different differentiated RNA under the induction of specific antibiotics [141]. As shown in Figure 7b, Liu and co‐workers et al. [142] analyzed the transcriptomes of *K. pneumoniae* strains with or without the resistance gene tmexCD‐toprJ using RNA sequencing and identified 12 differentially expressed RNAs in response to tigecycline. Based on this panel, they developed a corresponding reverse transcription‐PCR (RT‐PCR) assay that achieved >94% classification accuracy within 3 h. Similarly, Ismagilov and co‐workers et al. [143] focused on drug‐resistant *Neisseria gonorrhoeae*, a slow‐growing pathogen, and identified two significantly upregulated transcripts (porB and rpmB) within 5 min of exposure. These markers were subsequently validated via digital PCR, enabling accurate resistance classification in 49 clinical isolates within 10 min.

Until now, rapid RNA biomarker‐based assays have been used to detect a wide range of bacterial susceptibility to antibiotics, including fluoroquinolones, aminoglycosides, carbapenems, tigecycline, and colistin, using RNA sequence and PCR [16, 140, 142, 144]. Complementing the instrument technologies, emerging point‐of‐care platforms, such as CRISPR/Cas and Argonaute systems, now revolutionize RNA detection [145]. These platforms precisely recognize pathogen‐specific RNA targets, activating collateral cleavage of reporter probes to generate quantifiable signals, demonstrating significant potential for next‐generation AST. Although RNA markers offer a rapid response and do not require prolonged incubation, their detection depends on specialized platforms and prior knowledge of bacterial species and their differentially expressed RNA [140].

#### 3.3.2 Differentially Expressed Metabolites

Antibiotic exposure over several hours perturbs bacterial metabolism in both resistant and susceptible strains. Susceptible bacteria typically undergo rapid metabolic collapse, characterized by the disruption of key biosynthetic pathways. In contrast, resistant strains can withstand antibiotic pressure but often acquire resistance‐conferring mutations that impose a fitness cost. This fitness burden drives compensatory physiological processes, leading to distinct alterations in metabolic profiles [146]. Disentangling these differential responses not only provides mechanistic insight into antibiotic action but also uncovers molecular biomarkers for rapid phenotypic AST.

Building on this concept, metabolomics has emerged as a powerful approach for detecting antibiotic‐induced metabolic shifts. By qualitatively and quantitatively profiling small‐molecule metabolites, metabolomics provides a comprehensive view of bacterial physiological states. Most metabolomics approaches aim to capture a broad spectrum of metabolites, although full structural identification is often limited [147]. Therefore, metabolomics‐based AST typically relies on advanced analytical techniques such as MS, Fourier‐transform infrared (FT‐IR) spectroscopy, and surface‐enhanced Raman scattering (SERS). For example, Pomázi and co‐workers et al. [148] analyzed FT‐IR bands corresponding to key biomolecules, including membrane fatty acids (3000–2800 cm^−1^), protein amide bands (1800–1500 cm^−1^), and polysaccharides (1200–900 cm^−1^), to differentiate between resistant and susceptible phenotypes. Umapathy and co‐workers et al. [149] used Raman spectroscopy to distinguish resistant and susceptible strains of *E. coli*, *Acinetobacter baumannii* (*A. baumannii*), and *Enterobacter spp*, before and after exposure to antibiotics with different modes of action, including ciprofloxacin, gentamicin, meropenem, and nitrofurantoin. Spectral changes in nucleic acid‐, protein‐, and lipid‐associated bands displayed opposite trends between resistant and susceptible strains, enabling accurate classification (Figure 7c).

Beyond untargeted metabolic fingerprinting, some studies emphasize targeted quantification of specific metabolites with high structural resolution, which are mechanistically associated with resistance or susceptibility [65, 150]. A notable example is purine metabolism during the stringent response, a common bacterial defense triggered by nutrient deprivation or antibiotic stress. In this process, the intracellular alarmone (p)ppGpp orchestrates transcriptional reprogramming by downregulating growth‐related genes and upregulating stress‐response pathways [151, 152]. This process involves the degradation of rRNA and tRNA, resulting in the rapid release (within 30 min) of purine degradation products into the extracellular environment, primarily adenine, guanine, xanthine, and hypoxanthine [153, 154]. Wang and co‐workers et al. [154] revealed that individual *Staphylococcus aureus* (*S. aureus*) and *E. coli* could release millions of adenine and hypoxanthine molecules into aqueous environments within 1 h, confirming their potential as rapid AST biomarkers. Furthermore, since bacteria also display purine‐associated vibrational signatures on their cell surfaces, SERS detection after short antibiotic incubation can reveal metabolic activity without requiring supernatant analysis. Ziegler and co‐workers et al. [155] observed the secretion of purines by analyzing the SERS spectra of viable bacterial cells excited with 785 nm laser radiation. Building on this finding, their team developed a SERS platform capable of determining the MIC within 1 h by comparing signal changes before and after antibiotic treatment under 785 nm excitation.

Beyond purines, other secondary metabolites also reflect resistance phenotypes. Vikesland and co‐workers et al. [156] showed that ampicillin‐exposed, resistant *P. aeruginosa* produced elevated levels of the redox‐active metabolite pyocyanin. In addition, phenazine‐1‐carboxylic acid and ovoflavin have similarly demonstrated potential as metabolic biomarkers of antibiotic susceptibility [157]. Taken together, metabolic alterations offer powerful insights into the biochemical basis of antibiotic response, enabling the development of rapid phenotype‐based AST methods. With continued advancements in analytical technologies, standardization of protocols, and integration with other omics data, metabolomics is poised to play a central role in the next generation of rapid, mechanism‐informed AST platforms.

### 3.4 Enzymatic Function‐Driven Biomarkers

Bacteria naturally secrete a variety of enzymes to sustain their metabolic activity and environmental adaptability. However, upon exposure to effective antibiotics, susceptible strains often undergo cell death or dormancy, resulting in a significant reduction in enzyme secretion. In contrast, resistant bacteria may actively secrete antibiotic‐inactivating enzymes as part of their defense mechanisms. Therefore, monitoring specific enzyme activities offers a promising phenotypic approach for rapid AST.

#### 3.4.1 Catalase Activities

**FIGURE 8:**
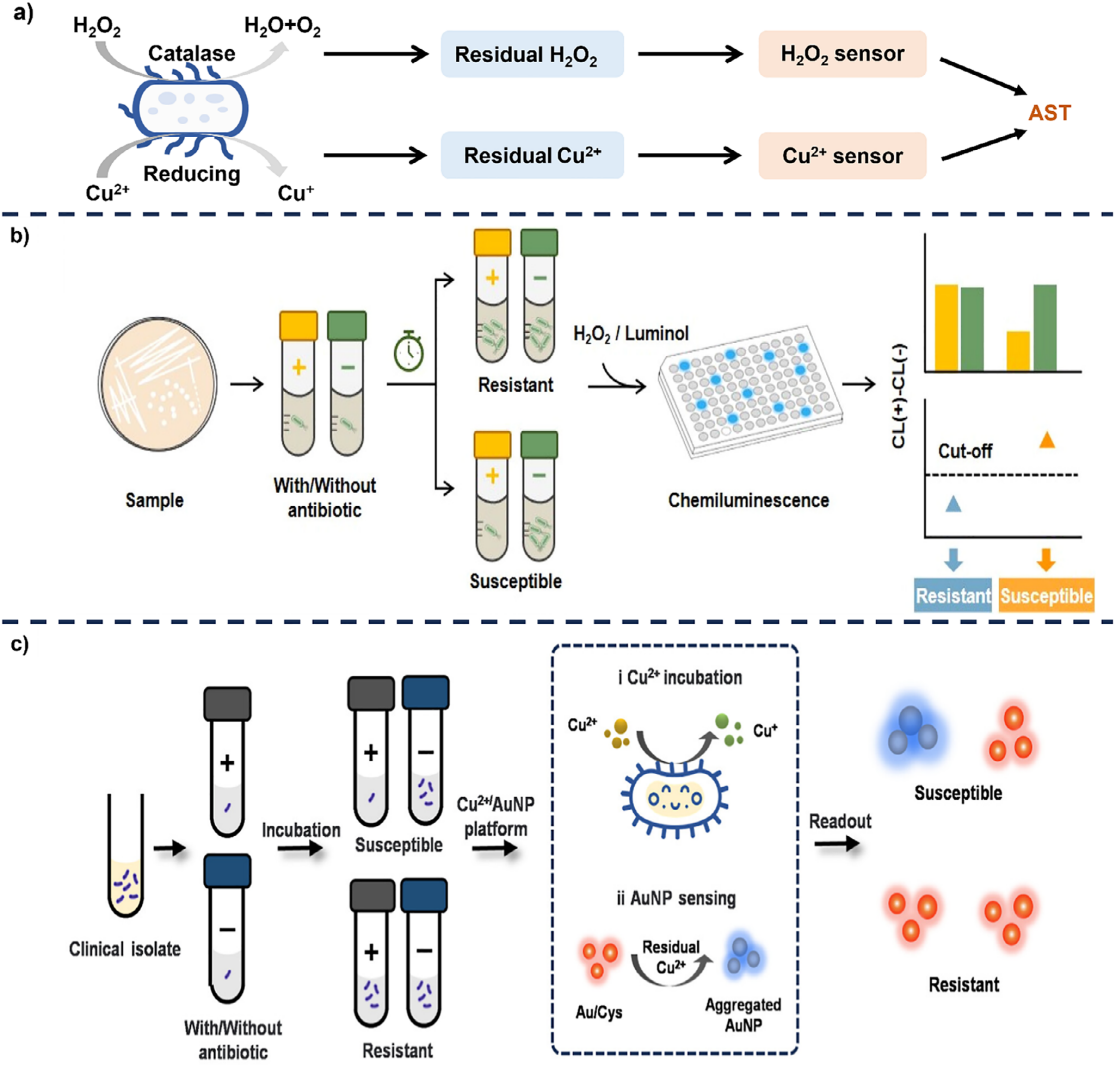
AST platform based on the catalase activities and the Cu^2+^‐reducing activity. (a) Principle of AST sensors leveraging bacterial catalase and Cu^2+^‐reducing capabilities. (b) Principle of a catalase–H_2_O_2_ chemiluminescence sensor‐based AST developed by Chen and co‐workers et al. Bacterial catalase catalyzes the decomposition of H_2_O_2_, modulating luminol‐induced chemiluminescence. The AST outcome (susceptible or resistant) is determined by the chemiluminescence difference between control and antibiotic‐treated samples. Reproduced with permission [168], Copyright 2022, American Chemical Society. (c) Principle of an AST strategy based on the Cu^2+^–Au/Cys platform developed by Wang and co‐workers et al. Cu^2+^ induces aggregation of Au/Cys nanoparticles, resulting in color changes. For the resistant strain, Cu^2+^ is reduced, inhibiting aggregation and producing a red solution with dispersed Au/Cys, enabling AST. Reproduced with permission [176], Copyright 2024, American Chemical Society.

Catalase plays an essential role in maintaining bacterial physiological activity by mitigating oxidative stress. It is widely expressed across bacterial species and found on the surface of some bacterial membranes, including those of *E. coli, S. aureus, K. pneumoniae, A. baumannii, and P. aeruginosa* [158]. It decomposes hydrogen peroxide (H_2_O_2_) into water and oxygen (Figure 8a). Leveraging this enzymatic activity. Various phenotypic AST have been developed for catalase‐positive bacteria, in which bacteria are incubated with H_2_O_2_ and the residues are quantitatively detected [159]. Detection methods are diverse and include chemiluminescence [160], colorimetry [161], and electrochemical sensing [162]. To enhance detection sensitivity and signal diversity, a variety of nanomaterials with peroxidase‐mimicking activity have been incorporated into biosensing platforms. These include fluorescent carbon‐based materials [163, 164], metallic nanomaterials [165, 166], and single‐atom nanozymes [167]. As shown in Figure 8b, Chen and co‐workers et al. [168] developed a chemiluminescence (CL) biosensor utilizing luminol for rapid and accurate AST of *E. coli and S. aureus*. In this system, luminol produced CL in the presence of H_2_O_2_. Antibiotic‐resistant bacteria efficiently decomposed H_2_O_2_, leading to reduced residues in the medium and diminished CL intensity. Consequently, the susceptibility of bacteria can be quantitatively assessed by comparing the CL intensity of the antibiotic‐treated and control groups. In a similar strategy, Chen and co‐workers et al. [166] utilized platinum nanoparticles with intrinsic peroxidase‐like activity to construct an electrochemical platform for detecting residual H_2_O_2_ in AST assays. Chen and co‐workers et al. [169] further developed a sensor array by exploiting residual H_2_O_2_ to mediate AuNPs synthesis and generate colorimetric signals. In this system, bacterial catalase‐mediated H_2_O_2_ consumption produced species‐specific metabolic fingerprints, combined with machine learning, enabling bacterial identification and AST of clinical isolates with an overall accuracy of 97.62%. However, the applicability of catalase‐based AST may be limited for catalase‐negative strains or when using bacteriostatic antibiotics that do not significantly alter bacterial viability.

#### 3.4.2 Cu^2+^‐Reducing Activity

Copper (Cu) is an essential trace element for all living organisms, serving as a critical cofactor in key biological processes, including aerobic respiration and superoxide dismutation [170]. To maintain intracellular homeostasis, most bacteria possess intrinsic Cu^2+^‐reducing activity to regulate intracellular copper levels [171]. They reduce toxic Cu^2+^ to less harmful Cu^+^ through reductases (such as CueO and CopA), while utilizing efflux pumps to expel or store Cu^+^ (Figure 8a). This inherent redox activity has been harnessed for rapid AST, most notably through Cu^+^‐catalyzed click chemistry, which provides a robust and selective readout of bacterial viability [172, 173]. Under reductive conditions, Cu^2+^ is converted in situ to catalytically active Cu^+^, which facilitates the formation of thermodynamically stable 1,4‐disubstituted 1,2,3‐triazoles between terminal alkynes and azides, forming a strong binding. For instance, Wang and co‐workers et al. [174] developed a click chemistry‐based platform in which CuCl_2_ and alkyne‐functionalized gold nanorods (AuNRs) were co‐incubated with bacterial cultures. Then, the mixture was introduced onto azide‐functionalized coverslips and incubated at 37°C for 2 h. The resistant bacteria effectively reduced Cu^2+^ to Cu^+2^, triggering the click reaction and immobilizing AuNRs onto the substrate. The quantity of surface‐bound AuNRs was subsequently visualized using dark‐field microscopy, enabling AST within 3 h. Similarly, Chen and co‐workers et al. [175] designed a chemical sensor utilizing the click chemical reaction for rapid AST.

Beyond click chemistry, copper‐mediated reactions have also been adapted for phenotypic AST. As cysteine residues on copper chaperones bind and transport copper ions, Gao and co‐workers et al. [176] synthesized cysteine‐modified gold nanoparticles (AuNPs‐cys) to establish a colorimetric AST platform. For susceptible strains, Cu^2+^ remained unreduced, inducing aggregation of AuNPs‐cys and resulting in a visible color change. This enabled the rapid determination of bacterial susceptibility within approximately 3 h (Figure 8c).

#### 3.4.3 Other Functional Enzyme Activity

**FIGURE 9:**
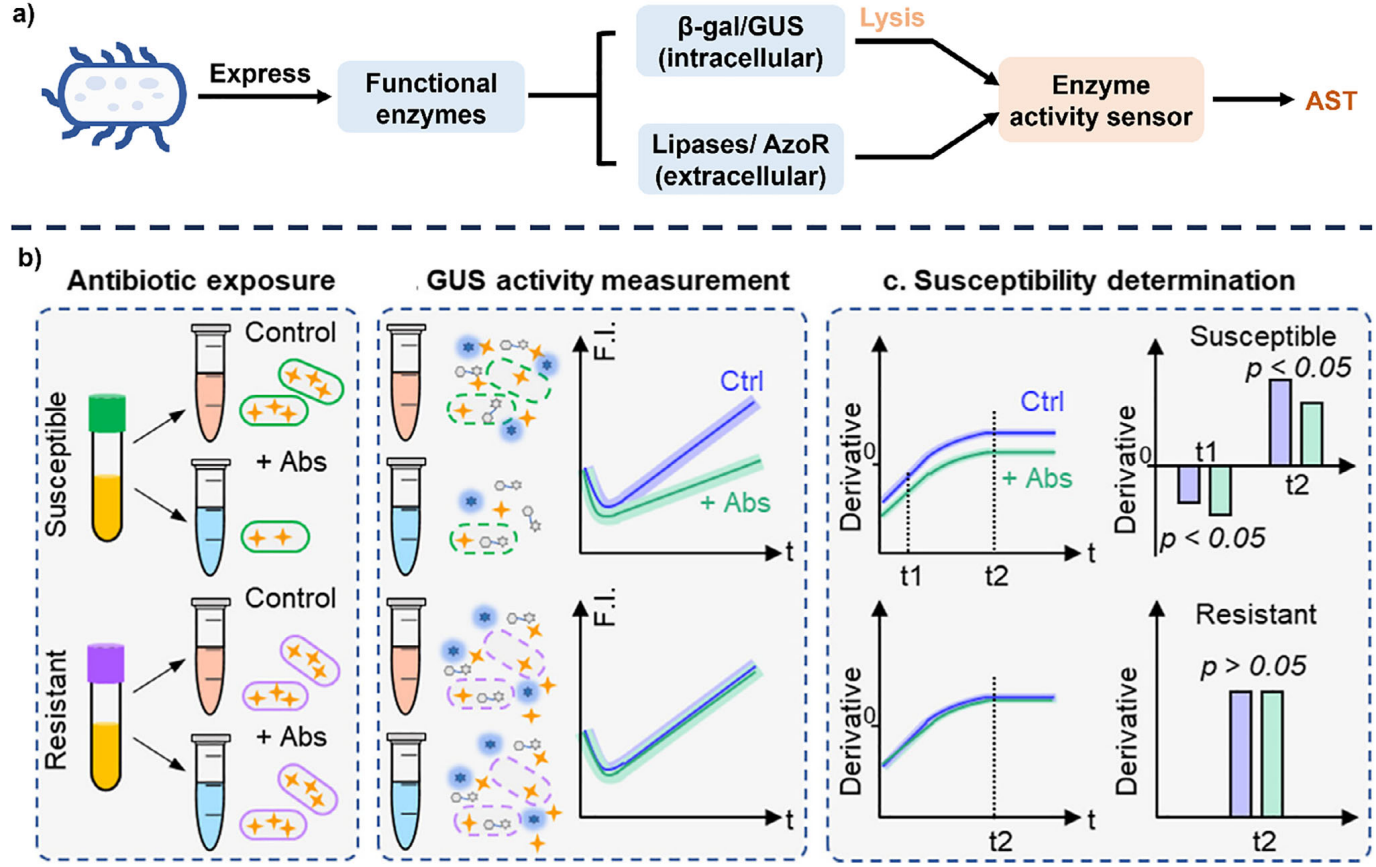
AST platform based on the activity of other functional enzymes. (a) Principle of AST strategies utilizing the activities of essential bacterial enzymes, such as glycosidases and lipases. Antibiotic‐susceptible bacteria show inhibited growth and decreased secretion of essential enzymes when exposed to antibiotics, while resistant strains maintain enzyme secretion. (b) Principle of the GUS‐based AST developed by Mu and co‐workers et al. In susceptible *E. coli* exposed to antibiotics, GUS expression was significantly reduced compared to untreated controls, resulting in a lower increase in fluorescence intensity after cell lysis. Conversely, resistant *E. coli* maintained GUS expression at levels similar to controls, leading to minimal differences in enzyme activity between treated and untreated groups, indicating antibiotic resistance. Reproduced with permission [178], Copyright 2023, American Chemical Society.

Bacteria produce a wide array of functional enzymes both intracellularly and extracellularly. Many of these enzymes are universally and abundantly expressed, making them intuitive and real‐time biomarkers of bacterial viability (Figure 9a). In addition, their well‐defined catalytic sites enable straightforward detection with substrate‐based sensors. Among them, β‐galactosidase (β‐gal) is a well‐characterized hydrolase that catalyzes the hydrolysis of β‐galactosides and is crucial for lactose metabolism. It has been widely used as a reporter enzyme due to its high expression in *E. coli* [159]. Leveraging this feature, Xiao and co‐workers et al. [177] developed a dual‐mode fluorescent and colorimetric platform based on β‐gal activity to differentiate susceptible and resistant *E. coli* strains upon antibiotic exposure. In antibiotic‐susceptible strains, β‐gal expression was significantly repressed during drug exposure, whereas resistant strains continued to produce. Similarly, β‐glucuronidase (GUS), a glycosidase involved in the metabolism of β‐D‐glucuronic acid, is prevalent in Enterobacteriaceae, particularly in *E. coli*, and has been employed as a molecular marker in microbial detection. As shown in Figure 9b, Mu and co‐workers et al. [178] demonstrated its utility as a dynamic AST biomarker for *E. coli* and *K. pneumoniae*. Real‐time enzymatic kinetics were quantified by the first derivative of fluorescence intensity, with the earliest significant difference between treated and control groups (*p* < 0.05) defined as t1. A second time point, t2, was determined when the reaction reached a steady rate. The presence of t1 and a persistent difference at t2 indicated susceptibility, whereas their absence suggested resistance.

Beyond glycosidases, other extracellular enzymes such as lipases and azoreductases (AoZR), enabling AST without bacterial lysing. Lipases contribute to lipid degradation and membrane remodeling, while AoZR reduces azo bonds using NAD(P)H as a cofactor. To harness these activities, Wang and co‐workers et al. [13] designed a dual‐probe system targeting the AoZR and lipase activity. The platform incorporated two distinct fluorophores, each quenched by a specific partner, and functionalized to respond to different target enzymes. Upon enzyme‐specific cleavage, the corresponding linkage between the fluorophore and quencher was disrupted, releasing fluorescence. This selective activation allowed simultaneous monitoring of both enzymes in a single assay. With machine learning‐assisted analysis, the platform could determine *E. coli* and *S. aureus* susceptibility to various antibiotics within 5 h. In general, identifying highly expressed, functionally relevant enzymes across bacterial species offers a promising strategy for developing rapid and sensitive AST methods.

#### 3.4.4 Antibiotic Inactive Enzymes

**FIGURE 10:**
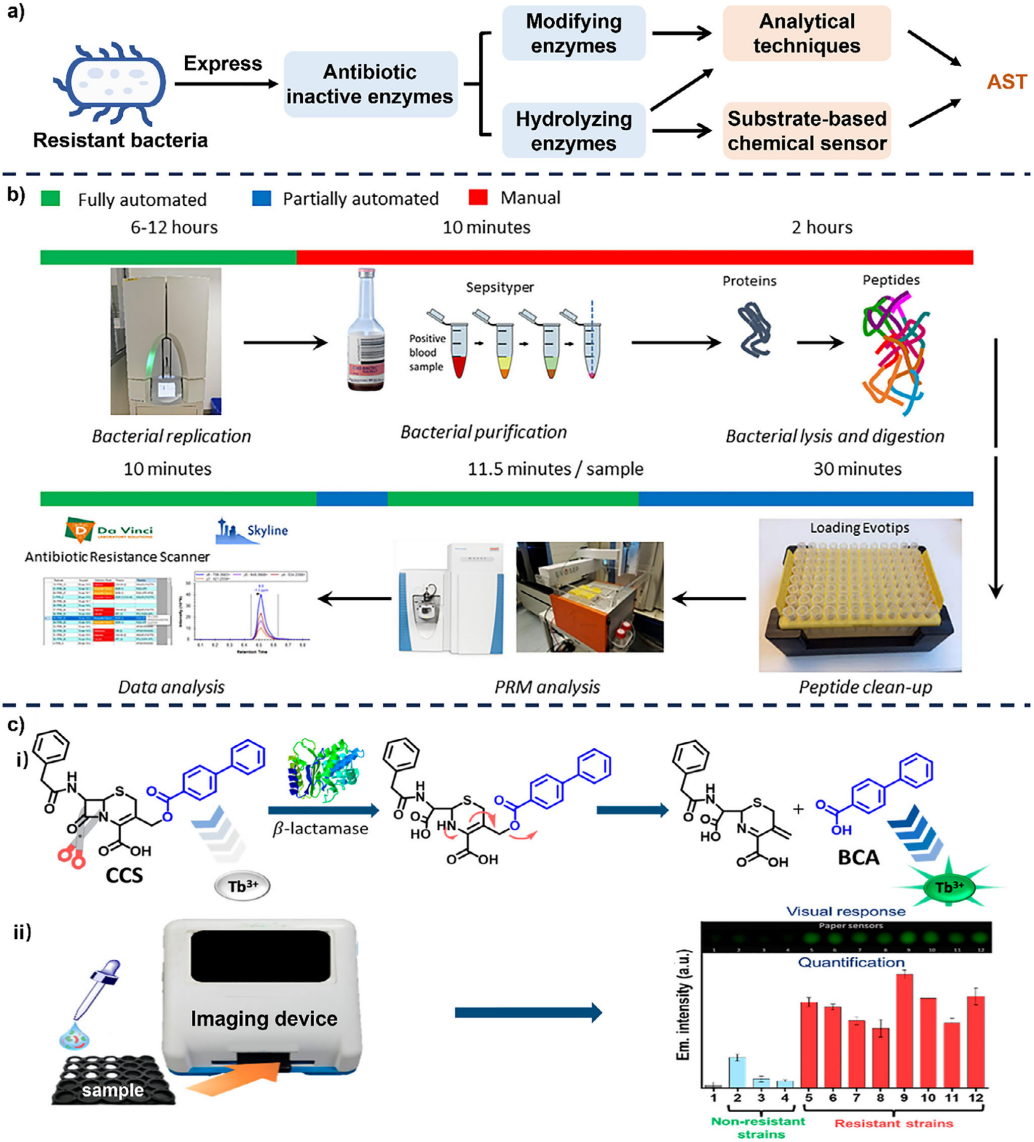
AST platform based on inactive antibiotic enzymes. (a) Principle of AST strategies targeting specialized enzymes expressed by resistant bacteria, including modification enzymes and antibiotic‐hydrolyzing enzymes. Modification enzymes alter antibiotics or their targets, while hydrolyzing enzymes directly cleave antibiotics, both leading to loss of antibiotic efficacy. (b) Principle of the targeted LC‐MS/MS‐based AST developed by Goessens and co‐workers et al. Resistance‐associated proteins were identified by detecting selected specific peptides, with SIL peptides enabling automated analysis. Reproduced with permission [182], under CC BY license. PRM: parallel reaction monitoring. (c) Principle of the β‐lactamase‐based AST developed by Maitra and co‐workers et al. (i) β‐lactamase hydrolyzes the β‐lactam ring of its substrate, releasing BCA, which induces terbium ion (Tb^3+^) fluorescence. (ii) Fluorescence signals were recorded using an imaging device for qualitative AST determination. Adapted with permission [189], Copyright 2024, American Chemical Society.

The production of antibiotic‐inactivating enzymes is one of the key resistance mechanisms employed by bacteria, making them valuable phenotypic biomarkers for AST, including modifying enzymes and antibiotic‐hydrolyzing enzymes (Figure 10a). Modifying enzymes alter the structures of antibiotic or their target sites through chemical modifications such as acetylation or phosphorylation, rendering the antibiotics ineffective. Owing to the structural complexity of these enzymes, it is often necessary to identify characteristic peptide fragments, which are then detected using advanced analytical techniques. Dekker and co‐workers et al. [179] developed a rapid targeted LC‐MS/MS approach to detect aminoglycoside‐modifying enzymes and 16S rRNA methyltransferases in *E. coli* and *K. pneumoniae*, enabling resistance profiling against gentamicin, tobramycin, and amikacin within 3.5 h. This method used proteogenomic techniques to select peptide candidates corresponding to modifying enzymes and incorporated internal quality control peptides to ensure proper sample preparation. Bacterial proteins are extracted via cell lysis and digested into peptides for LC‐MS/MS detection. Due to its multiplexing capability, this strategy holds promise by integrating additional peptides to simultaneously detect multiple inactive antibiotic enzymes [180, 181].

Similarly, as shown in Figure 10b, Goessens and co‐workers et al. [182], using a targeted LC‐MS/MS method, detected 27 clinically significant resistance‐associated proteins in blood cultures positive for *E. coli* or *K. pneumoniae*. This comprehensive approach covered key resistance mechanisms to β‐lactams, aminoglycosides, and fluoroquinolones, including 10 β‐lactamases, 7 aminoglycoside‐modifying enzymes, 4 16S rRNA methyltransferases, 4 DNA gyrase‐related proteins, and 2 porins. Meanwhile, the researchers carefully optimized stable isotope‐labeled (SIL) peptide concentrations to approximate endogenous peptide levels, while establishing a standardized detection protocol to ensure automated and reproducible peptide quantification prior to clinical validation.

In addition to modifying enzymes, bacteria produce antibiotic‐hydrolyzing enzymes that directly cleave essential chemical structures in antibiotic molecules, resulting in irreversible inactivation and high‐level resistance. β‐lactamases are the most clinically significant, hydrolyzing the β‐lactam ring of penicillin, cephalosporins, monobactams, and carbapenems. This hydrolysis prevents the combination of PBPs, thus representing a major cause of β‐lactam resistance in Gram‐negative bacteria [183]. According to the β‐Lactamase Database (http://www.bldb.eu/; last accessed May 19, 2025), over 11 534 β‐lactamases have been identified to date and are categorized into four major classes (A–D) based on sequence homology. Based on the spectrum of β‐lactam hydrolysis, β‐lactamases can be classified into four major groups: broad‐spectrum β‐lactamase (BSBL), extended‐spectrum β‐lactamase (ESBL), *AmpC* β‐lactamase (AmpC), and carbapenemases [184]. Their detection can also be accomplished using instrumental analysis methods. However, due to their well‐characterized active sites and ability to be secreted extracellularly, β‐lactamases are well‐suited for phenotypic detection strategies.

A wide range of chromogenic and fluorogenic substrates has been developed to allow simple, often naked‐eye detection based on color or fluorescence changes induced by β‐lactam ring hydrolysis [185]. These changes arise from molecular rearrangements and shifts in electron distribution upon ring cleavage [186]. For instance, nitrocefin, a type of commercial substrate, was developed by Glaxo in 1971 and remains widely used [40]. Especially, Liu and co‐workers et al. [187] first showed that nitrocefin and its analogues exhibit enhanced Raman scattering after β‐lactamase‐catalyzed hydrolysis. On this basis, they developed a stimulus‐responsive resonance Raman scattering strategy for the rapid in situ detection and imaging of β‐lactamase‐producing bacteria with single‐cell sensitivity. This chromogenic cephalosporin undergoes a distinct color shift from yellow to red upon β‐lactam ring hydrolysis. However, its high cost and synthesis complexity limit widespread use. Additionally, the more predominant method of substrate designs is combining a detection module with a probe unit, where enzymatic cleavage of the β‐lactam ring triggers probe liberation to generate detectable signals [188]. For example, Maitra and co‐workers et al. [189] rationally designed a cephalosporin‐based probe (CCS) that remains weakly emissive in its intact form. Upon hydrolysis by β‐lactamase, the probe releases a terbium‐sensitizing moiety (BCA), producing a strong luminescence signal (Figure 10c). This “turn‐on” signal features large Stokes shifts and long emission lifetimes, enabling highly sensitive detection.

The assays based on one type of substrate often suffer from poor specificity and sensitivity, particularly when detecting carbapenemases, resulting in potential false negatives [17]. Liu and co‐workers et al. [190] developed an ultrasensitive carbapenemases‐specific chromogenic probe by optimizing electron‐withdrawing substituents and identifying −N^+^(CH_3_)_3_ as the optimal group. Integrated with a portable paper chip, this platform enabled direct detection of carbapenemases‐producing bacteria in sputum within 15 min, with 100% clinical sensitivity and specificity across 80 clinical samples. Refining substrate specificity could enable β‐lactamase subtype differentiation, allowing personalized antibiotic therapy and better antimicrobial stewardship. Furthermore, Liu and co‐workers et al. [184] developed a visual sensor system incorporating four modified chromogenic substrates, each tailored to one of the four major β‐lactamase subtypes (BSBL, ESBL, AmpC, and carbapenemases), 1–2 orders of magnitude more sensitive than conventional probes. Also, the sensor can provide results within 0.25–3 h, achieving a detection limit as low as 10^4^ CFU mL^−1^, and contribute to rapid and precise medicine. The immunochromatographic assays also represent a rapid and cost‐effective alternative for the detection of β‐lactamase [17]. However, bacteria harbor multiple resistance mechanisms, such as efflux pumps or bypass pathways. Therefore, the absence of antibiotic‐inactivating enzymes does not guarantee susceptibility.

## 4 Comparison and Translational Limitations

The above categorizes the existing metabolic biomarkers and outlines some developments in metabolism‐based AST. The representative approaches are summarized in Table 2. Although these studies demonstrate the promise of metabolic responses for rapid phenotypic AST, they differ in detection biomarker, sample type, readout technology, and practical applicability. To better understand this field, we will compare these strategies in detail and consider their limitations in clinical translation to provide useful insights for future development.

**TABLE 2:** Overview of the emerging metabolic‐based biomarkers and technologies for rapid phenotypic AST.

AST biomarker	AST technology	Sample type	Bacteria type	Bacteria concentration	Time to result	Advantages	Ref.
Glucose consumption	MALDI‐TOF‐MS	Identified colonies from blood cultures	*E. coli* and *Klebsiella* spp.	—	90–150 min	Able to be integrated into a clinical laboratory	[89]
	IR‐ATR	Bacterial suspension	*E. coli*	10^9^ CFU mL^−1^	2 h	Continuously record to improve the quality of AST	[91]
Glucose/H_2_O utilize	MALDI‐MS	Bacterial suspension	*E. coli*	2 × 10^8^ CFU mL^−1^	2 h	Require a small volume of bacteria culture and antibiotic/D_2_O	[96]
	Raman scattering	Bacterial suspension	*E. coli, K. pneumoniae, S. aureus*	OD_600_ = 0.1	10 min–1 h	Ultra‐rapid metabolic imaging based on single‐cell, which is useful for nonculturable or fastidious bacteria	[92, 94, 95]
	Wide‐field mid‐infrared imaging	Bacterial suspension	*E. coli*	5 × 10^5^ CFU mL^−1^	1 h	Quantify protein synthesis at the single‐cell level	[93]
D‐AA incorporation	Colorimetric Sensor	Bacterial suspension or spiked serum	*E. coli, S. aureus, etc*.	10^5^–10^6^ CFU mL^−1^	6 h	Discriminate bacteria and AST using the specific metabolic features of bacteria to D‐AA	[97]
	Fluorescent imaging	Bacterial suspension	*E. coli and B. subtilis*	OD_600_ = 0.4–0.6	10 min–2 h	Visualize the bacterial response to drugs, providing insight into bacterial cell morphology	[99, 100]
pH variation	Microfluidics	Bacterial suspension	*Lactobacillus crispatus*	∼10^6^ CFU mL^−1^	20–40 min	Enables rapid monitoring of bacterial metabolism and quantitative MIC estimation	[103]
	Electrical impedance spectroscopy	Bacterial suspension	*E. coli, K. pneumoniae, S. aureus, etc*.	∼10^6^ CFU mL^−1^	1 h	Simple, label‐free, and suitable for integration into clinical workflow	[108]
	Colorimetric Sensor	Bacterial suspension	*K. pneumoniae, A. baumannii, E. coli*	∼10^7^ CFU mL^−1^	2 h	Cost‐effective, biocompatible, naked‐eye detection and digital image analysis for semi‐quantitative results	[15, 109]
	Electrochemical pH sensing	Bacterial suspension	*E. coli, Streptococcus salivarius*	∼10^5^ CFU mL^−1^	∼1 h	Avoids interference from dead bacteria and provides accurate MIC estimation	[104]
Oxygen consumption	Oxygen electrode sensor	Immobilized bacteria on the electrode	*M. tuberculosis*	∼10^7^ CFU mL^−1^	4 days	Sensitive to minute changes in respiration and can detect multiple drugs	[116]
	Optical oxygen‐sensing	Bacterial suspension	*E. coli*	∼5 × 10^5^ CFU mL^−1^	2–3 h	Real‐time monitor oxygen consumption rate for MIC determination	[115, 118]
	polydopamine Sensor	Bacterial suspension	*E. coli*	1.7 × 10^5^– 2.7 × 10^5^ CFU mL^−1^	3–5 h	Detect antibiotic resistance via oxygen‐dependent dopamine polymerization, visual readout	[117]
	Nanosensors integrated into biofilm	Biofilm	*P. aeruginosa*	Biofilm from OD_600_ = 0.05 inoculum	6 h	Reflects biofilm phenotype, dynamic metabolic response	[119]
Extracellular electron transfer	Disposable electrochemical sensor	Bacterial suspension	*S. maltophilia, S. sonnei*,	10^7^–10^8^ CFU mL^−1^	4–5 h	Portable, cost‐effective, disposable	[125]
	Electrochemical sensor	Bacterial suspension	*P. aeruginosa, E. coli, S. aureus, S. saprophyticus*	∼10^8^–10^9^ CFU mL^−1^	96min; 30 min	Label‐free, all‐electrical, suitable for integration into clinical workflow	[12, 14]
	Colorimetric Sensor	Bacterial suspension	*S. aureus, K. pneumoniae, P. aeruginosa; E. coli*	OD_600_ = 0.1–0.3; 5 × 10^2^–10^7^ CFU mL^−1^	2–4 h	Simple, visual readout, works for non‐electrogenic bacteria	[126, 129]
	Paper‐based microfluidics	Bacterial suspension	*E. coli*, *P. aeruginosa, S. aureus, etc*.	∼1.5 × 10^8^ CFU mL^−1^	8–15 h	Ultra‐low cost, multi‐antibiotic and multi‐bacteria testing; disposable	[128]
ATP level	Colorimetric Sensor	Blood samples; environmental samples	*E. coli, S. aureus, A. baumannii, K. pneumoniae*	≥10 CFU mL^−1^; 2 CFU mL^−1^	∼5 min; ∼90 min	High‐throughput, smartphone‐based analysis	[135, 136]
	Bioluminescence sensor	Bacterial suspension	*Enterobacterales*	∼1.5 × 10^8^ CFU mL^−1^	∼3 h	Rapid, visual luminescence readout, no cell lysis required	[139]
Differentially expressed RNA	RT‐PCR	Bacterial suspension	*K. pneumoniae*	≥10^6^ CFU mL^−1^	3 h	Simultaneous genotype and phenotype detection, >94% accuracy, detect efflux pump‐mediated resistance	[142]
	Multiplexed hybridization‐based RNA detection	Positive blood cultures	*K. pneumoniae, E. coli, A. baumannii, S. aureus, P. aeruginosa*	≥10^7^ CFU mL^−1^	<4 h	Combines phenotypic and genotypic AST in one assay, works across multiple antibiotic classes, highly accurate (94%–99%)	[140]
Metabolomics	FT‐IR + MALDI‐TOF MS	Identified colonies from yogurt	*Lactobacillus, Lacticaseibacillus, S. hermophilus*	—	∼48 h	Rapid phenotypic typing, useful for food safety monitoring	[148]
	Raman Spectroscopy	Bacterial suspension	*E. coli*, *A. baumannii*, *Enterobacter* spp.	OD_600_ = 0.8	6–8 h	Label‐free, able to integrate machine learning for prediction	[149]
Pyocyanin level	SERS	Bacterial suspension	*P. aeruginosa, E. coli*	∼10^8^ CFU mL^−1^	< 24 h	Reveal metabolite‐mediated resistance, real‐time monitoring	[156]
Purines level	SERS	Bacterial suspension	*E. coli, K. pneumoniae, E. faecium, S. saprophyticus*	∼10^6^ CFU mL^−1^	∼1 h	Ultra‐rapid MIC determination, label‐free	[155]
	Microfluidics‐integrated SERS	Bacterial suspension	*E. coli, B. subtilis*	5 × 10^8^ CFU mL^−1^	∼30 min	High‐resolution discrimination of similar strains	[153]
Catalase activity	Plasmonic nanosensor	Urine samples	*E. coli, K. pneumoniae*	∼10^6^ CFU mL^−1^	85–100 min	On‐site, no lab equipment, smartphone integration, high accuracy	[159]
	Electrochemical sensor	Bacterial suspension	*E. coli, S. aureus*	∼10^7^ CFU mL^−1^	45–60 min	High sensitivity, low cost (<$0.1/test), reusable	[166]
	Chemiluminescence sensor	Urine samples	*E. coli, K. pneumoniae*	≥5 × 10^5^ CFU mL^−1^	∼40 min	Direct‐from‐urine AST, self‐calibration avoids inoculum bias, high accuracy	[165]
Cu^2+^‐reducing activity	Click chemistry‐based sensor	*Plasma, urine, blood* samples	*E. coli;*	∼10^6^, 10^1^–10^7^ CFU mL^−1^	∼60 min; ∼3 h	High sensitivity, portable	[174, 175]
	Plasmonic nanosensor	Bacterial suspension	*E. coli, S. aureus*	10^3^–10^7^ CFU mL^−1^	∼3 h	Naked‐eye readout, smartphone integration, low cost	[176]
GUS activity	Fluorescent enzymatic sensor	Urine samples	*E. coli, K. pneumoniae*	∼6–8 × 10^5^ CFU mL^−1^	25–60 min	MIC determination, broad antibiotic coverage	[178]
β‐gal activity	Fluorescence/colorimetric dual‐mode sensor	Bacteria‐spiked food samples	*E. coli*	10^2^–10^5^ CFU mL^−1^	∼4–5 h	Portable, smartphone‐assisted, visual readout, low‐cost	[177]
AzoR and lipase activity	Luminescence sensor	Bacterial suspension	*S. aureus, S. epidermidis, E. coli, etc*.	OD_600_ ≈ 1	∼5 h	Integrate machine learning for bacteria identification and AST, high accuracy, and MIC estimation	[13]
Aminoglycoside‐modifying enzymes	Targeted LC‐MS/MS	Bacterial suspension	*E. coli, K. pneumoniae*	OD_600_≈ 1	∼3 h	High sensitivity and specificity, multiplex detection, and able to predict phenotypic resistance	[179]
β‐lactamase	LC‐MS/MS	Bacterial suspension	*A. baumannii, K. pneumoniae etc*.	—	∼90 min	High sensitivity and specificity	[180]
	Colorimetric paper‐based sensor	Diverse body fluids (sputum, urine, blood, etc.)	*E. coli, K. pneumoniae, M. morganii*	∼10^4^ CFU/mL	0.25–3 h	Low‐cost, portable, subtype‐level identification	[184, 185]

### 4.1 Comparative Assessment of Biomarkers and Technologies

From the perspective of biomarker selection, biomarkers related to nutrient uptake or respiratory activity generally provide the broad phenotypic coverage [92, 102]. Changes in glucose consumption, H_2_O utilization, D‐amino acid incorporation, oxygen, and ATP consumption all indicate whether bacteria remain metabolically active after antibiotic exposure. These activities are usually common in bacteria, allowing the corresponding assays to be applied across a broad range of species [93]. Instead, they convert bacterial activity into measurable chemical signals, making them readily compatible with established analytical methods for AST [89, 90]. However, these approaches often still require an incubation period before metabolic differences become distinguishable, as bacteria need time to shift from an active state to growth arrest, dormancy, or metabolic suppression under effective antibiotic treatment [97, 116]. In most cases, these biomarkers reflect global physiological activity rather than the underlying cause of resistance. Their readouts can be influenced by growth phase, oxygen conditions, and medium composition, thereby affecting AST accuracy [191]. As a result, although these biomarkers are often sensitive to early antibiotic effects, their specificity may vary across bacterial species and testing conditions.

Metabolic reprogramming‐based biomarkers generally provide higher biological resolution. Differentially expressed RNA and metabolite fingerprints can capture how bacteria reorganize metabolic pathways under antibiotic stress, thereby offering a closer view of the adaptive responses [140]. In addition, transcriptional changes are among the earliest cellular responses under stress, giving RNA an intrinsic advantage for rapid AST [16]. Metabolomic profiling can reveal coordinated changes across multiple molecular features within one assay. However, the higher information content of this category is accompanied by reduced universality. These approaches often require prior knowledge of the bacterial species, corresponding biomarkers, and complex sample pretreatment, while they usually depend on advanced instrumentation and data interpretation [142, 149].

Enzymatic function‐driven biomarkers offer a distinct advantage because enzyme‐catalyzed reactions can amplify signals, making them well‐suited to optical readouts and portable platforms in clinical sample [159]. For example, the AST of catalase‐positive bacteria can be converted into the sensitive detection of residual H_2_O_2_ using established biosensors. Assays targeting common functional enzymes, such as GUS, β‐gal, and lipase, may also avoid prior pathogen identification [13]. However, they are limited in practice as many of these enzymes are intracellular and expressed at low levels, requiring bacterial lysis before detection [177]. Antibiotic‐inactivating enzymes, such as β‐lactamases and aminoglycoside‐modifying enzymes, offer stronger mechanistic specificity. Yet their scope is inherently narrow, because they cover only defined resistance mechanisms [182]. Enzyme expression also varies across bacterial species, so prior pathogen identification is still needed to determine whether a given enzyme is a relevant target. Among these, for some extracellular enzymes, it is possible to detect bacteria in the clinical sample directly [17, 174, 175].

Different analytical technologies are not equally suitable for all metabolic biomarkers. In general, for single chemical species or enzymes, such as glucose, ATP, GUS, β‐gal, and lipase, electrochemical, colorimetric, fluorescent, and luminescent sensors are the most practical. These methods are less reliant on complex instrumentation, easier to miniaturize, lower in cost, and more compatible with point‐of‐care deployment [192]. Meanwhile, they avoid complicated spectral interpretation and provide intuitive signal outputs. However, many of these methods rely on bulk metabolic changes and often require relatively high bacterial concentrations or prior enrichment to generate detectable signals. In many cases, they do not require complex sample pretreatment, but their performance may also be influenced by background interference in complex clinical samples [193, 194].

In contrast, vibrational spectroscopy (such as SERS) and mass spectrometry provide richer molecular information and capture more subtle metabolic changes for phenotypic analysis [195]. These approaches are particularly valuable for metabolism‐based AST because they can resolve multidimensional biochemical signatures rather than relying on a single metabolite or enzymatic product [147]. This gives them stronger analytical depth and higher sensitivity for AST [146]. However, these advantages are accompanied by higher instrument cost, operational complexity, and centralized laboratories [196]. RNA‐based molecular approaches can also provide rapid and highly sensitive readout, as transcriptional changes occur early after antibiotic exposure. However, unlike broader physiological readouts, they depend on prior pathogen identification and pre‐established species‐specific biomarkers [142].

Above all, no single metabolism‐based AST strategy is universally optimal. Biomarkers reflecting broad physiological activity are useful for rapid phenotypic screening without prior bacterial identification, but provide lower sensitivity and specificity. In contrast, metabolic reprogramming‐ and some enzyme‐based readouts may offer improved mechanistic relevance but come at the expense of generalizability, simplicity, or instrument reliance. Similarly, simpler readout platforms may enable lower‐cost, whereas instrument platforms provide more information. The value of an analytical platform should not be judged by time‐to‐result alone, but by considering detection requirement, cost, complexity, need for prior identification, and applicability across different bacterial types. Therefore, the selection of metabolism‐based AST strategies should be guided by the intended application scenario, the bacterial species and sample type, and the balance between mechanistic information and practical implementation. To further strengthen this comparison, we added Tables 3 and 4 to summarize the comparative features of the major metabolic biomarker categories and the representative analytical platforms, respectively.

**TABLE 3:** Comparative assessment of the four major metabolic biomarker categories in metabolism‐based AST.

Category	Representative readout	Early response	Mechanistic relevance	Time to result	Species applicability	Sample applicability	Advantages	Limitations	Ref.
Nutrient uptake	Glucose consumption, H_2_O utilization, D‐AA incorporation	Detectable after short incubation	Low	Hours	Broad	Limited in polymicrobial samples	Broad phenotypic coverage, compatible with established analytical readouts	Reflects bulk metabolic activity, easily affected by polymicrobial samples, sometimes requires complex preprocessing	[89, 94, 96, 97, 98, 99]
Respiratory activity	pH variation, oxygen consumption, extracellular electron transfer, ATP turnover	Detectable after short incubation	Low	Hours to days	Physiology dependent	Limited in polymicrobial samples	Rapid physiological response, suitable for real‐time and portable detection	Easily affected by the environment; difficult to assign in polymicrobial samples	[115, 116, 117, 118, 135, 136]
Metabolic reprogramming	Differentially expressed RNA and metabolites	Rapid, especially for RNA	High	Minutes to hours	Dependent on pre‐established species‐specific biomarkers	Requires sample pretreatment	Higher biological resolution, closer insight into adaptive responses	Often species‐ or antibiotic‐dependent, requires pre‐selected biomarkers, complex preprocessing	[140, 141, 142, 148, 149]
Enzymatic function	Catalase, GUS, β‐gal, lipase, Cu^II^‐reducing activity, β‐lactamase	Rapid after substrate conversion	Variable; high for resistance enzymes	Minutes to hours	Physiology dependent	Clinical sample including plasma, urine, blood	Signal amplification through catalysis, some targets provide strong mechanistic relevance	Depending on enzyme abundance and accessibility, some targets require lysis, resistance enzymes cover only limited mechanisms	[13, 165, 166, 177, 178, 179, 184]

**TABLE 4:** Comparative assessment of representative analytical approaches in metabolism‐based AST.

Category	Representative technology	Time to result	Detection requirement	Cost	Complexity	Ref.
Electrochemical sensor	Electrochemical pH, oxygen, and redox assays	Minutes to days	Often require high bacterial input or incubation, some assays require prior identification	Low to moderate	Low to moderate	[12, 14, 104, 116, 125, 166]
Colorimetric sensor	pH, D‐AA, and enzyme‐responsive assay	Hours	Often require incubation, some assays require bacterial lysis and prior identification	Low	Low	[97, 109, 184, 185]
Luminescence sensor	Luciferase‐based ATP and enzyme‐catalyzed assay	Minutes to hours	Often require incubation, some assays require bacterial lysis and prior identification	Low	Low	[139, 165, 178]
Vibrational spectroscopy	SERS, FT‐IR, and IR‐ATR	Hours	Often require purified bacterial suspensions, controlled matrices, and prior identification	High	High	[91, 92, 94, 95, 155, 156]
Mass spectrometry	MALDI‐MS, LC‐MS/MS	Hours	Require purified bacteria, isolated colonies, processed samples, and prior identification	High	High	[89, 96, 148, 180]

### 4.2 Limitations in Broader Diagnostic Workflow

Although many metabolism‐based AST platforms can generate rapid signals after antibiotic exposure, their practical value should be considered within the diagnostic workflow rather than at the sensing step alone. In clinical practice, the total turnaround time is determined not only by how quickly a metabolic response can be measured, but also by how rapidly a clinically relevant bacterial population can be obtained from the original specimen [97]. From this perspective, many currently reported metabolism‐based AST methods mainly shorten the interval between antibiotic challenge and phenotypic readout, but do not yet eliminate the upstream dependence on bacterial isolation, enrichment, or inoculum standardization. This limitation is evident from the studies summarized in Table 2. Most current methods have been demonstrated using cultured isolates, purified bacterial suspensions, or spiked monocultures in artificial media rather than direct clinical specimens. This experimental design is understandable as metabolic signals will be influenced by bacterial concentration, growth phase, and matrix composition [169]. The biosensors are easier to control under laboratory conditions. However, it means that the assay times reported for many metabolism‐based AST methods do not directly correspond to the time required from specimen collection to a clinical result.

A major reason for this gap is that clinical samples are inherently more complex than artificial media. In blood, urine, sputum, wound exudates, or other biofluids, pathogens may be present at low abundance and coexist with host cells, proteins, salts, and endogenous metabolites [197]. These factors can interfere with metabolism‐based AST in several ways. First, low bacterial abundance may produce signals below the effective detection range, especially for approaches based on bulk changes in nutrient consumption or secreted enzyme activity [198]. Second, host‐derived metabolites can increase the background signal to affect the baseline and reduce the contrast between susceptible and resistant strains [199]. As a result, additional culturing, enrichment, or sample‐processing steps are required before testing, diminishing the advantage of a rapid metabolic readout.

Another limitation is that most reported metabolism‐based AST studies are based on monoculture systems, whereas clinical infections involve multiple species. In polymicrobial samples, different bacterial species may consume overlapping substrates, release similar metabolites, or generate mixed enzymatic signals, making the readout less specific [200]. Moreover, interspecies interactions, such as cross‐feeding or enzyme‐mediated protection, may cause the measured response to deviate from the intrinsic susceptibility of the target pathogen [201]. Under these conditions, direct measurement of the sample reflects total metabolic activity rather than the antibiotic response of the target pathogen alone. This issue is particularly important for broad physiological biomarkers, whose main advantage is species independence. In mixed infections, different bacterial species may consume or release overlapping substrates and generate combined signals, making the results more difficult to interpret.

These limitations help explain why, compared with traditional phenotypic platforms and genotypic assays, relatively few metabolism‐based AST products have reached commercialization, as the advantage of rapid metabolic sensing is often offset by upstream sample‐processing requirements and the difficulty of maintaining robust, standardized performance across diverse bacterial samples and polymicrobial clinical conditions.

## 5 Conclusion

This review outlined the classic antibiotic target and the resistance mechanisms, emphasizing the metabolic difference they bring. Understanding these shifts not only provided biological insight but also pointed to potential targets for metabolism‐based AST strategies. We summarized the emerging metabolic biomarkers applied in rapid phenotypic AST, categorizing them according to their association with nutrient uptake, respiratory activity, metabolic reprogramming, and enzymatic function. In addition, we introduced the corresponding detection strategies for each biomarker. An overview of representative studies is summarized in Table 2, while Tables 3 and 4 provide a critical comparison of metabolic biomarker categories and analytical platforms, respectively. Metabolism‐based biomarkers are reshaping our understanding of AST, providing a dynamic window into how bacteria respond to antibiotic pressure. Looking ahead, as knowledge of bacterial metabolic responses deepens and integrated methods continue to develop, this area is well placed to move from mechanistic studies toward routine clinical application, supporting more rational antimicrobial use and helping to curb resistance

## 6 Future Perspectives

Metabolism‐based biomarkers represent a promising direction for rapid phenotypic AST. By capturing the early metabolic perturbations, they provide a dynamic readout of bacterial response. Despite initial progress, several challenges remain in identifying robust biomarkers and translating metabolism‐based AST into clinically actionable systems.

### 6.1 Expanding the Metabolic Biomarker Landscape

Current metabolism‐based AST approaches rely on a limited range of biomarkers, ranging from altered metabolite levels to resistance‐associated enzymes. Many of these biomarkers exhibit antibiotic‐ or species‐specific responses, which constrain their general applicability, while pathogens may harbor multiple resistance mechanisms or require prior pathogen identification. Furthermore, bacterial resistance continues to evolve, with new resistance determinants emerging alongside the development of novel antimicrobial agents. To address these limitations, future efforts should focus on expanding the metabolic biomarker landscape. Integrating multi‐omics approaches, including metabolomics, transcriptomics, and proteomics, may facilitate the identification of more conserved metabolic responses that reflect fundamental bacterial adaptation to antimicrobial stress. Such broad‐spectrum biomarkers could enable universal AST without requiring detailed prior bacterial identification.

### 6.2 Linking Metabolism to Mechanism

While expanding biomarker coverage is essential, increased breadth alone is insufficient without mechanistic clarity. At present, many reported metabolic biomarkers remain correlative, reflecting downstream stress responses rather than direct consequences of antibiotic action or resistance mechanisms, limiting diagnostic specificity. Further efforts are needed to establish clearer connections between metabolic responses and underlying resistance mechanisms. For example, elevated levels of antibiotic‐modifying enzymes, altered precursor flux in cell wall biosynthesis, or shifts in central metabolism may reflect specific adaptive strategies employed by resistant bacteria. Strengthening the mechanistic understanding of such metabolic signatures could improve diagnostic interpretability and support the rational development of future antimicrobial and diagnostic strategies.

### 6.3 Translational Challenges for Clinical Diagnostics

Translating metabolism‐based AST from laboratory studies to routine clinical diagnostics remains challenging. Compared with traditional phenotypic AST and genotypic resistance tests, metabolism‐based AST measures physiological responses rather than stable growth endpoints or defined genetic targets, which will be influenced by bacterial species, antibiotic class, or sample matrix [191]. Many current metabolism‐based AST studies still rely on pure bacterial suspensions and mainly accelerate post‐isolation readout rather than provide true sample‐to‐answer susceptibility testing. For clinical translation, the key issue is not only requiring short turnaround times but also producing reliable results. Therefore, only a few metabolism‐based AST products have made it to commercialization.

To move beyond this limitation, future development should focus on integrated workflows rather than isolated sensing modules [198, 202]. A practical route is to combine metabolism‐based AST with upstream pathogen capture and matrix reduction rather than optimizing sensing chemistry alone, including selective bacterial enrichment, host‐cell depletion, microfluidic concentration, and analysis at the single‐cell or small‐population level. These steps are important for improving analytical sensitivity, reducing background interference, and improving species attribution in mixed samples. For complex specimens, metabolism‐based readouts may also need to be coupled with parallel pathogen identification so that the signal can be interpreted in the target bacteria. In addition, metabolism‐based AST systems must be validated against CLSI and EUCAST with acceptable categorical agreement, reproducibility, and error rates. Such validation should extend beyond ideal laboratory conditions to clinical settings, including polymicrobial samples and complex biofluids. Commercial translation will also require standardized workflows and reproducible performance across laboratories and instruments. Without solving sample processing, standardization, and reproducibility together, faster metabolic sensing alone is unlikely to translate into clinical application.

### 6.4 Point‐of‐Care Testing and Data‐Driven Analysis

Point‐of‐care testing (POCT) may help address several translational barriers associated with metabolism‐based AST. Advances in microfluidics, lab‐on‐chip platforms, and rapid sample handling have been shown to accelerate bacterial metabolic activity by 2–5 fold, enabling earlier detection of metabolic changes [203]. When combined with portable detection modalities, such as fluorescence‐based readouts, smartphone imaging, or paper‐based microfluidic devices, these systems could facilitate rapid and cost‐effective AST outside centralized laboratories.

Importantly, while POCT platforms improve accessibility and reduce turnaround time, the metabolism‐based AST typically reflects multidimensional metabolic responses, arising from the combined effects of multiple metabolic features, temporal dynamics, and context‐dependent variables. These readouts may include mass spectrometry spectra, SERS fingerprints, isotope labeling patterns, or multiplexed biomarker signals, which are difficult to interpret using conventional threshold‐based analysis. In this context, artificial intelligence (AI) and machine learning (ML) approaches offer a systematic means to integrate multiple metabolic features and account for their combined contributions.

First, AI‐assisted analysis is useful for extracting susceptible data from complex signals. Rather than relying on a single peak or endpoint, ML can integrate multi‐dimensional spectral and morphological features to identify subtle changes that are not easily resolved by manual analysis [195]. This is particularly relevant for metabolism‐based AST, where antibiotic response is often distributed across multiple correlated variables. Second, ML is useful for increasing practical robustness. In real samples, metabolic signals can vary with bacterial physiology, sample matrix, and instrument settings. Models trained on diverse datasets may help compensate for such variability by learning invariance [204]. This is especially valuable for portable platforms, where environmental variation is harder to control than in centralized laboratories. Third, future metabolism‐based AST systems can expand the collection of signals from multiple biomarkers, time points, or sensing modalities. Beyond binary classification, AI may enable the detailed definition of metabolic responses. For example, instead of reporting only susceptible or resistant, AI‐based analysis may support the estimation of resistance levels or early MIC‐related trends from continuous metabolic changes, or even pathogen identification [205, 206]. More importantly, AI and ML should be viewed as a complement rather than a substitute for biomarker design and robust workflow integration. To achieve clinically reliable performance, models must be developed using datasets with different bacterial species, antibiotics, sample matrices, and instrument conditions.

### 6.5 Toward a Structured AMR Framework for Metabolism‐Based AST

Despite progress in biomarker discovery, device development, and data analysis, a structured AMR framework for metabolism‐based AST has not yet been fully established. Currently, many metabolic biomarkers can only distinguish between drug‐resistant and drug‐sensitive states, failing to differentiate between “low‐level resistance” and “high‐level resistance,” and cannot be equated with MIC values to reflect the actual degree of drug resistance. For metabolism‐based AST to support clinical decision‐making, it is important to clarify how specific metabolic biomarkers and detection methods relate to resistance against specific antibiotic classes or subclasses, whether they can distinguish narrow‐spectrum resistance from multidrug resistance, and to what extent metabolic responses correlate with clinically relevant MIC ranges. Without such definitions, metabolic signals may be difficult to interpret beyond general susceptibility or resistance classification. Future research should focus on developing a performance metric system that clarifies the applicable range of bacterial species, the antibiotic spectrum covered, and how biomarker concentrations correspond to resistance levels under clinically relevant conditions, such as low bacterial loads and complex sample matrices.

## Author Contributions

**Sha Yu**: writing – original draft, writing – review and editing, visualization. **Bayinqiaoge**: writing – review and editing. **Xi Lu**: writing – review and editing. **Xin Wang**: writing – review and editing. **Rongfeng Wang**: writing – review and editing. **Yi Li**: writing – review and editing. **Shi‐Yang Tang**: writing – review and editing, conceptualization. **Chengchen Zhang**: writing – review and editing, conceptualization, visualization, funding acquisition.

## Funding

This work was supported by EPSRC New Investigator Award (APP34994), UK Research and Innovation (UKRI), UK; Wessex Medical Innovation Fund (AF06), Wessex Medical Trust, UK; Standard Research Grant (RG∖R1∖241228) and International Exchanges 2023 (IEC∖NSFC∖233339) & 2025 (IES∖R2∖252009), The Royal Society, UK.

## Conflicts of Interest

The authors declare no conflicts of interest.

## Data Availability

Data sharing not applicable to this article as no datasets were generated or analyzed during the current study.
